# Effects of caffeine intake on exercise performance in basketball players: a systematic review and meta-analysis

**DOI:** 10.3389/fnut.2026.1837912

**Published:** 2026-05-07

**Authors:** Jingxin Liu, Ruiguo Xue, Xuanjia Zhang, Jin Huang, Bin Chen, Li Guo, Yinhang Cao, Olivier Girard

**Affiliations:** 1 School of Physical Education, Shanghai University of Sport, Shanghai, China; 2 School of Athletic Performance, Shanghai University of Sport, Shanghai, China; 3 Department of Public Physical Education, Fujian Agriculture and Forestry University, Fuzhou, China; 4 School of Exercise and Health, Shanghai University of Sport, Shanghai, China; 5 School of Human Sciences (Exercise and Sport Science), The University of Western Australia, Perth, Australia

**Keywords:** basketball performance, caffeine supplementation, ergogenic aid, physical performance, team sports, technical skills

## Abstract

**Background:**

The ergogenic effects of caffeine in team sports, particularly basketball, have been widely investigated. This systematic review and meta-analysis evaluated the effects of caffeine intake on basketball-specific skills and general physical and game-related performance outcomes.

**Methods:**

Eighteen studies employing a blinded, crossover design were included. Random-effects meta-analyses examined the effects of caffeine intake on isolated skills (e.g., free-throw and three-point shooting accuracy, dribbling speed), general physical performance (e.g., sprint speed, jump height, and agility), and game-related actions during real or simulated matches (e.g., assists, total points scored, and performance index rating).

**Results:**

Low to moderate doses of caffeine intake improved general physical performance, including linear sprint speed (SMD: −0.27; 95% CI: −0.42– −0.13; *p* < 0.01), repeated-sprint speed (SMD: −0.45; 95% CI: −0.78 −0.13; *p* < 0.01), single-jump height (SMD: 0.15; 95% CI: 0.01–0.29; *p* = 0.04), and agility, reflected by shorter test completion times (SMD: −0.25; 95% CI: −0.51–0.00; *p* = 0.05). Low caffeine doses (2.3–3 mg/kg) did not significantly improve basketball-specific skills (i.e., shooting accuracy and dribbling speed), but were associated with enhanced overall performance during real or simulated competition (SMD: 0.25; 95% CI: 0.10–0.40; *p* < 0.01), alongside increased perceived muscle endurance (SMD: 0.53; 95% CI: 0.12–0.95; *p* = 0.01) and power (SMD: 1.01; 95% CI: 0.63–1.39; *p* < 0.01).

**Conclusion:**

In basketball, low-dose caffeine primarily enhances general physical rather than isolated technical skills. These findings highlight the importance of dose selection, suggesting that moderate caffeine dosage (>3 mg/kg) may be required to meaningfully influence performance in match-like basketball settings.

**Systematic review registration:**

https://www.crd.york.ac.uk/PROSPERO/view/CRD420251060676, Identifier: CRD420251060676.

## 1 Introduction

Basketball is a globally practiced, high-intensity intermittent team sport characterized by frequent transitions between offensive and defensive phases, repeated player-player interactions, and continuous decision-making under physical and perceptual stress (1). Across a typical match (four quarters of 10–12 min), players repeatedly perform sport-specific technical actions (i.e., dribbling, shooting, passing, and rebounding) while executing rapid movements such as sprinting, accelerations and decelerations, and multidirectional changes of direction (2–4). Importantly, these technical actions must be executed with precision under conditions of cumulative neuromuscular fatigue and elevated cognitive load. Thus, competitive basketball performance depends not only on physical capacities, but also on the ability to preserve technical proficiency and decision-making accuracy throughout the game.

Caffeine (1,3,7-trimethylxanthine) is among the most extensively researched and widely consumed ergogenic aids in sport. A robust body of evidence supports its performance-enhancing effects across multiple exercise modalities, including endurance (5, 6), anaerobic (7), resistance (8), and sprint-based activities (9). These ergogenic effects are attributed to a combination of central and peripheral mechanisms such as antagonism of adenosine receptors, increased neural drive, altered perception of effort, and enhanced muscle contractility (10, 11). While the benefits of caffeine are well established in individual sports, growing interest has shifted toward its application in team sports, where performance arises from the interaction between physical output, technical execution, and tactical skills (12, 13).

Recent meta-analytic evidence indicates that caffeine supplementation enhances high-intensity actions (e.g., accelerations, decelerations, and body impacts) in intermittent sports including team, racket, and combat sports (13). However, this analysis did not examine sport-specific technical skills in team sport athletes. Addressing this limitation, a recent meta-analysis in volleyball players demonstrated that caffeine intake improves both physical performance and volleyball-specific actions, such as attack and serve accuracy, during competition (14). Although informative, substantial differences between volleyball and basketball in movement patterns, skill constraints, game tempo, and cognitive demands limit the extent to which caffeine-related effects can be extrapolated between the two sports.

To date, basketball-specific evidence remains fragmented. Two narrative reviews suggest that caffeine may enhance selected physical performance variables in basketball players, such as vertical jump ability and agility, but report inconsistent effects on basketball-specific skills, including shooting accuracy and dribbling speed (15, 16). In recent years, the number of original studies examining caffeine supplementation in basketball has increased (17–19), encompassing both laboratory-based performance tests and sport-specific assessments conducted under simulated or competition conditions. Consequently, an updated systematic review with meta-analysis focused specifically on basketball players is warranted to provide practitioners with robust, evidence-based guidance on the potential utility of caffeine supplementation in basketball settings.

Therefore, the aim of this study was to systematically review and meta-analyze the effects of caffeine intake on basketball performance, with particular emphasis on both basketball-specific skills and general performance outcomes.

## 2 Materials and methods

### 2.1 Literature search

This systematic review and meta-analysis was conducted following the Preferred Reporting Items for Systematic Reviews and Meta-Analysis (PRISMA) guidelines (20) and registered with the International Prospective Register of Systematic Reviews (PROSPERO) database (CRD420251060676). A comprehensive literature search was performed using a combination of Medical Subject Headings (MeSH) and free-text terms related to caffeine supplementation and basketball performance. The search strategy incorporated four key concepts: (1) (caffeine OR coffee OR 1,3,7-Trimethylxanthine) AND (2) (supplement OR supplementation OR ergogenic aid) AND (3) (basketball OR simulated sports OR team sports) AND (4) (performance OR athletic performance OR sports performance OR physical performance). Electronic searches were conducted in five databases (PubMed, Web of Science, SCOPUS, Google Scholar, and EBSCO) from database inception through December 2025. The literature search was performed independently by two authors (J.L. and X.Z.).

### 2.2 Study selection

The selection process was conducted by two independent investigators (J.L. and X.Z.), with discrepancies resolved by a third investigator (Y.C.). The inclusion criteria were established based on the participants, interventions, comparators, outcomes, and study design (PICOS) framework (21): (1) Population: trained basketball players; (2) Intervention: acute caffeine intake (i.e., capsule, gum, and caffeinated beverages), provided that the independent effect of caffeine could be isolated; (3) Comparator: placebo supplementation; (4) Outcome: basketball-specific tests: free-throw and three-point shooting accuracy, and dribbling speed; Non-specific tests: linear and repeated sprint, single and repeated jump, and agility; Game actions during real or simulated competition: assists, rebounds, turnovers, body impacts, free-throw, two-point and three-point shooting accuracy, total points, and performance index rating; Physiological and perceptual responses: heart rate, rating of perceived exertion, perceived fatigue, muscle endurance and power related measures; (5) Study design: randomized single- or double-blind crossover designs; Only original research studies published in English (i.e., not a conference abstract or review) were included.

### 2.3 Data extraction

Data were independently extracted from each included study using a standardized form. Extracted information included: (1) study details (first author, year of publication); (2) participant characteristics (sample size, age, sex, sports performance level, and habitual caffeine intake); (3) caffeine intake protocol (form, timing, and dosage); (41) exercise or testing protocol; and (5) main findings.

Performance outcomes were grouped into four pre-defined categories: (1) Basketball-specific tests (22): free-throw and three-point shooting accuracy, and sprint with dribbling; (2) Non-specific physical tests (23): linear and repeated sprint, single and repeated jump, and agility; (3) Game actions during real or simulated competition (24): assists, rebounds, turnovers, body impacts, free-throw, two-point and three-point shooting accuracy, total points scored, and performance index rating. Performance index rating is a composite metric commonly used to evaluate a player's overall match performance in basketball. It is calculated as: (points + total rebounds + assists + steals + blocks + fouls received) – (missed shots + turnovers + fouls committed) (4, 25) Physiological and perceptual responses: heart rate, perceived fatigue, muscle endurance, and power-related measures.

### 2.4 Assessment of methodological quality

Methodological quality and risk of bias were independently evaluated by two investigators (J.L. and R.X.) using the Cochrane Collaboration's risk-of-bias tool (RoB version 2.0, based in London, United Kingdom) (26). Any disagreements were resolved through discussion and, when necessary, consultation with a third reviewer (Y.C.). The tool evaluated bias across the following domains: randomization process, deviations from intended interventions, missing outcome data, outcome measurement, selection of the reported result and overall bias risk.

### 2.5 Statistical analyses

All statistical analyses were performed in R (version 4.5.2, R studio) using the meta and metafor packages. Random-effects models were applied to assess the effects of caffeine and placebo on all performance outcomes. Results were reported as standardized mean difference (SMD), 95% confidence interval (CI), and *p*-values. SMD was calculated using adjust Hedges'g, which incorporates a correction factor to adjust for small sample bias. Effect sizes were interpreted as trivial (< 0.20), small (0.20–0.49), moderate (0.50–0.79), or large (≥ 0.80). Statistical heterogeneity was quantified using the I^2^ statistic and classified as low (< 25%), moderate (25–50%), or high (> 50%) (27). Meta-regression analyses were conducted to examine whether caffeine dose and training years influenced outcomes (e.g., specific and non-specific tests, game actions during real or simulated competition). Potential publication bias was assessed via funnel plots and Egger's tests. For time-based outcomes (sprint with dribbling, sprint speed, and agility), lower values indicate better performance; therefore, negative values favor caffeine. In addition, following Cochrane recommendations (28), outcome data for perceived fatigue were directionally inverted so that higher values consistently reflected improved performance, allowing alignment with perceived endurance and power in subgroup analyses. Statistical significance was set at *p* < 0.05.

## 3 Results

### 3.1 Study characteristics

**Figure 1:**
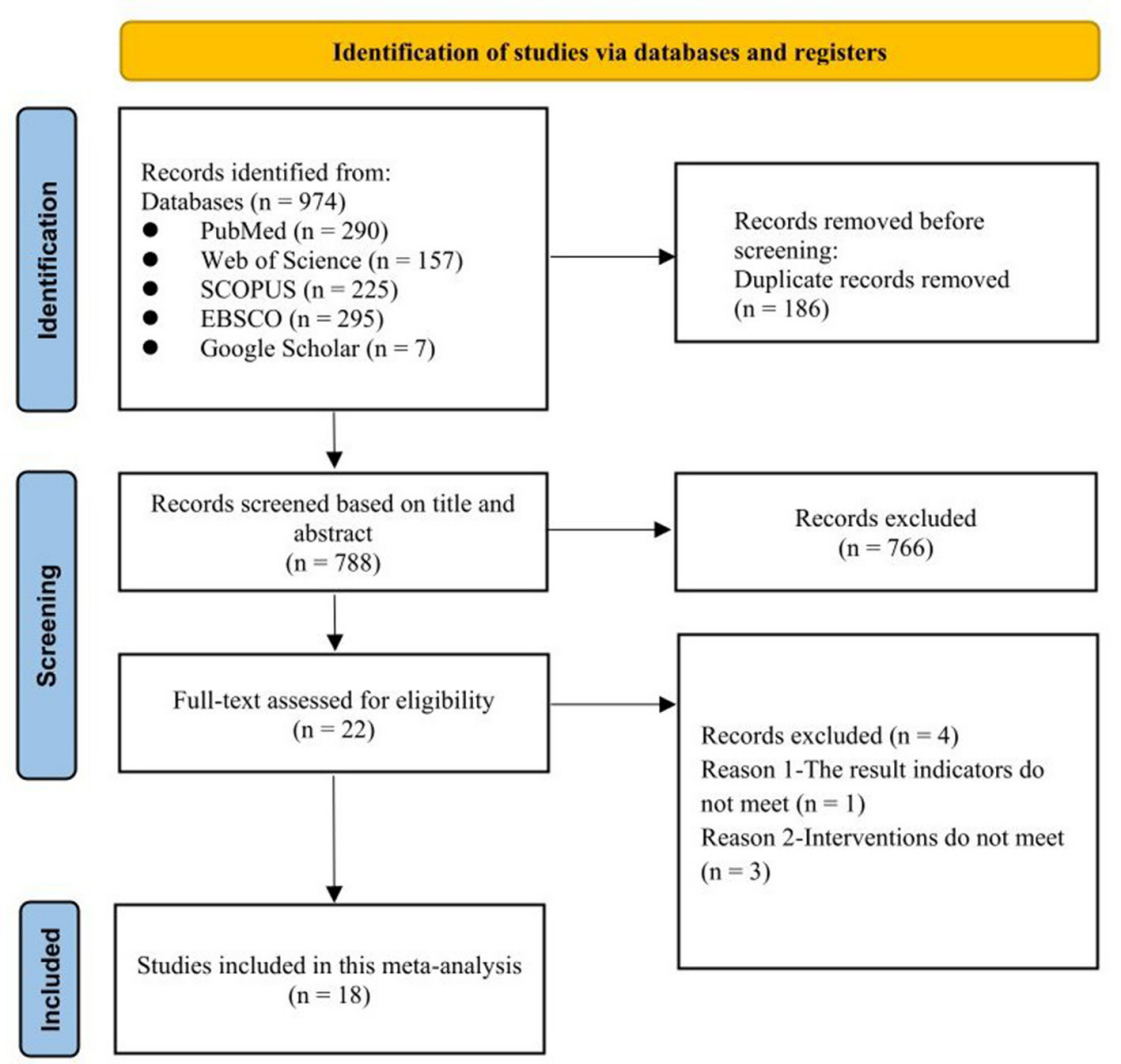
Flow diagram of study selection.

The study selection process is presented in Figure 1. A total of 974 studies were identified across databases. After removing duplicates, titles and abstracts were screened, resulting in 18 studies that met the eligibility criteria for analysis (2, 17–19, 29–42).

Table 1 summarizes the characteristics of the included studies, comprising a total of 288 basketball players (182 men, 106 women). According to the performance classification proposed by McKay et al. (43), 14 male participants were classified as *trained* or *developmental* players (tier 2), 226 participants (88 women) as *highly trained* or *national-level* players (tier 3), and 53 participants (19 women) as *elite* or *international-level* players (tier 4). Caffeine doses varied across studies, with fourteen studies administering 1–3 mg/kg and four studies using 4–6 mg/kg. Caffeine was most commonly provided in capsule form (11 studies), followed by caffeinated beverages (four studies), and chewing gum (three studies). In most studies (13 studies), caffeine was administered 60 min before exercise; however, several studies employed alternative timings, including 15 min (18, 31, 34), 70 min (42), or 80 min (17). Seventeen studies reported habitual caffeine intake, with most participants classified as low to mild caffeine consumers (i.e., 50–200 mg/d, 0.6–2.71 mg/kg/day) (44).

**Table 1:** Characteristics of the included studies.

Study	Sample + Age (years) Level + Training years	Habitual Caffeine Intake (mg/day)	Caffeine Form + Dose (mg/kg or mg) + Timing (min)	Comparator	Exercise protocol	Main findings
Pernigoni et al. (17)	14 M; 21 ± 2; Developmentally Trained; 12.9 + 1.9	217 ± 170	Capsule; 3; 80	Placebo (cellulose)	CMJ + Sprint (0 −20 m) test	→ CMJ (cm): (Pre: 46.6 ± 6 vs. 47 ± 5.6; Post: 44.1 ± 5.1 vs. 43 ± 5.1; 24 h post: 47.1 ± 5.7 vs. 47.1 ± 5.1), 10 m sprint (s): (Pre: 1.8 ± 0.1 vs. 1.8 ± 0.1; Post: 1.8 ± 0.1 vs. 1.8 ± 0.1; 24 h post: 1.8 ± 0.1 vs. 1.8 ± 0.1), 20 m sprint (s): (Pre: 3.1 ± 0.1 vs. 3.1 ± 0.1; Post: 3.1 ± 0.2 vs. 3.2 ± 0.2; 24 h post: 3.1 ± 0.2 vs. 3.1 ± 0.1), Perceived fatigue: (Pre: 2.7 ± 2.1 vs. 2.3 ± 1.2; Post: 4.1 ± 1.8 vs. 5.7 ± 2.3; 24 h post: 3.4 ± 1.7 vs. 3.4 ± 1.2).
Hemmat et al. (18)	20 M; 17 ± 1; Highly Trained; > 3	< 300	Gum; 3; 15	Placebo (decaffeinated gum)	VJ + V –COD + T –COD test	→ VJ (cm): (33.7 ± 4.6 vs. 34.4 ± 4.5), V –cut dribbling test (s): (10.7 ± 0.5 vs. 10.5 ± 0.7), T –COD (s): (11.1 ± 0.7 vs. 11.0 ± 0.7).
Nieto-Acevedo et al. (19)	12 F; 22 ± 4; Highly Trained; > 10	< 100	Capsule; 3; 60	Placebo (cellulose)	AJ + CMJ + AG (5 −0 −5) + Sprint (0 −20 m) + SM test	↑CMJ (cm): (27.9 ± 3.8 vs. 26.7 ± 3.2), AG (s): (2.7 ± 0.1 vs. 2.6 ± 0.2), AJ (cm): (32.1 ± 2.9 vs. 31.7 ± 2.9),Two –point shooting accuracy (%): (49.7 ± 22.6 vs. 26.9 ± 21.1); → Three –point shooting accuracy (%): (12.5 ± 22.6 vs. 4.2 ± 10.4), Free –throw accuracy (%): (16.7 ± 32.6 vs. 12.8 ± 20.3), Assists: (0.8 ± 1.2 vs. 1.0 ± 1.3), Total rebounds: (4.4 ± 3.1 vs. 4.3 ± 3.4), Turnovers: (1.5 ± 1.8 vs. 2.1 ± 1.2), Points: (6.6 ± 4.8 vs. 4.4 ± 3.6), Performance index rating: (7 ± 6 vs. 3 ± 7), Sprint: (3.3 ± 0.1 vs. 3.3 ± 0.1).
Tan et al. (29)	18 M; 24 ± 2; Highly Trained; > 3	< 200	Capsule; 3; 60	Placebo (maltodextrin)	VJ + Sprint (6 m) + Three –point shooting accuracy test	→ Three –point shooting accuracy (%): (CP: 12.6 ± 2.8; PC: 11.7 ± 3.8 vs. PP: 12.3 ± 4.1), VJ (cm): Q1 (CP: 63.7 ± 11.6; PC: 63.0 ± 8.3 vs. PP: 62.8 ± 8), Q2 (CP: 63.9 ± 11.9; PC: 63.5 ± 8.8 vs. PP: 62.5 ± 7.8), Q3 (CP: 63.4 ± 10.8; PC: 63.6 ± 7.7 vs. PP: 62.2 ± 7.4), Q4 (CP: 64.6 ± 10.4; PC: 64.8 ± 8.8 vs. PP: 62.9 ± 7.8), Sprint (s): Q1 (CP: 1.6 ± 0.19; PC: 1.6 ± 0.2 vs. PP: 1.6 ± 0.2), Q2 (CP: 1.7 ± 0.2; PC: 1.7 ± 0.2 vs. PP: 1.7 ± 0.2), Q3 (CP: 1.7 ± 0.2; PC: 1.7 ± 0.2 vs. PP: 1.8 ± 0.2), Q4 (CP: 1.7 ± 0.1; PC: 1.7 ± 0.2 vs. 1.8 ± 0.2).
Niknam et al. (30)	24 M; 21 ± 2; Highly Trained; > 2.5	< 229	Espresso; 80/160; 60	Placebo (decaffeinated coffee)	RST –TT test	↑RST –TT (s): (LDEC: 55.7 ± 2.3 vs. CON: 56.8 ± 2.8), Perceived fatigue (5 min after RST): (LDEC: 15.0 ± 7.5/HDEC: 15.9 ± 7.4 vs. CON: 17.8 ± 7.8); RST –TT (s): (HDEC: 54.6 ± 1.9 vs. CON: 56.8 ± 2.8), Perceived fatigue (immediately after RST): (LDEC: 59.0 ± 8.4; HDEC: 55.7 ± 8.7 vs. CON: 68.0 ± 12.8).
Liu et al. (31)	15 M; 21 ± 1; Highly Trained; 8.2 + 0.3	74 ± 24	Gum; 3; 15	Placebo (decaffeinated gum)	CMJ + AG + Sprint (0 −20 m) + Free –throw accuracy + RAST test	↑Free –throw accuracy (%): (79.0 ± 4.3 vs. 73.0 ± 9.2), Sprint fatigue index (%): (3.6 ± 1.6 vs. 5.2 ± 1.6), 10 m sprint (s): (1.6 ± 0.1 vs. 1.7 ± 0.1), 20 m sprint (s): (3.0 ± 0.1 vs. 3.1 ± 0.1); → CMJ (cm): (54.5 ± 4.7 vs. 56.5 ± 3.5), AG (s): (10.1 ± 0.4 vs. 10.2 ± 0.5).
Gasperi et al. (32)	8 M; 24 ± 6; Highly Trained; 13.5 + 5.6	N.A.	Capsule; 3; 60	Placebo (cellulose)	Real game test	→ PIR: (1 ± 0 vs. 1 ± 0), Points: (0.4 ± 0 vs. 0.4 ± 0), Rebounds: (0.2 ± 0.0 vs. 0.2 ± 0.0), Assists: (0.1 ± 0.0 vs. 0.1 ± 0.0), Turnovers: (0.1 ± 0.0 vs. 0.1 ± 0.0), Perception of stamina (6.2 ± 0.3 vs. 6.21 ± 0.3), Perception of power: (6.3± 0.2 vs. 6.1 ± 0.2).
Stojanovic et al. (33)	11 M; 17 ± 1; Highly Trained; 5.3 + 3.0	310 ± 76	Capsule; 3; 60	Placebo (dextrose)	CMJ + CMJAS + SJ + LAD + Sprint (0 −20 m) + Suicide run dribbling test	↑CMJ (cm): (morning: 33.9 ± 5.4 vs. 31.0 ± 5.0; evening: 33.9 ± 6.1 vs. 33.6 ± 5.8); → CMJAS (cm): (morning: 42.3 ± 5.7 vs. 40.0 ± 5.2; evening: 42.2 ± 6.1 vs. 42.9 ± 6.0), SJ (cm): (morning: 33.2 ± 4.7 vs. 30.6 ± 4.9; evening: 32.3 ± 5.1 vs. 31.7 ± 6.4), LAD (s): (morning: 12.0 ± 0.7 vs. 12.5 ± 0.8; evening: 12.6 ± 0.8 vs. 12.6 ± 0.9); → 5 m Sprint (s): (morning: 1.2 ± 0.1 vs. 1.2 ± 0.1; evening: 1.1 ± 0.1 vs. 1.1 ± 0.1), 10 m sprint(s): (morning: 2.0 ± 0.1 vs. 2.0 ± 0.1; evening: 1.9 ± 0.1 vs. 1.9 ± 0.1), 20 m sprint(s): (morning: 3.3 ± 0.2 vs. 3.4 ± 0.2; evening: 3.3 ± 0.2 vs. 3.3 ± 0.2), Suicide run dribbling (s): (morning: 28.5 ± 2.1 vs. 29.0 ± 2.3; evening: 32.7 ± 2.0 vs.32.4 ± 1.9).
Filip –Stachnik et al. (34)	9 F; 24 ± 4; Highly Trained; 11 + 3	154	Gum; 2.3 ± 0.2; 15	Placebo (decaffeinated gum)	Sprint (0 −20 m) + AG + CMJ + Free –throw + Three –point shooting accuracy test	→ Sprint (s): (5 m: 1.1 ± 0.0 vs. 1.1 ± 0.1; 10 m: 1.9 ± 0.1 vs. 1.9 ± 0.1; 20 m: 3.3 ± 0.1 vs. 3.4 ± 0.2), AG (s): (6.1 ± 0.3 vs. 6.1 ± 0.2), CMJ (cm): (32.8 ± 2.3 vs. 32.1 ± 2.3), Free –throw accuracy (%): (79.2 ± 12.5 vs. 75.0 ± 12.5), Three –point shooting accuracy (%): (60.0 ± 10.0 vs. 60.0 ± 10.0).
Raya–González et al. (35)	14 M; 21 ± 2; Elite; 6.8 + 1.1	100	Liquid; 6; 60	Placebo (sucrose)	Sprint (0 −20 m) + CMJ + LAD + RS (5 x 30 m) test	↑CMJ (cm): (39.5 ± 5.3 vs. 37.1 ± 5.1), Sprint (s): (3.1± 0.2 vs. 3.2 ± 0.2), LAD (s): (11.6 ± 0.6 vs. 12.1 ± 0.8), RSA (s): (33.5± 1.3 vs. 35.2 ± 1.6).
Tan et al. (36)	12 M/6 F; 23 ± 2; Highly Trained; > 3	< 200	Powder; 6; 60	Placebo (maltodextrin)	SM test	↑Heart rate (bpm): (set 1: 159 ± 12 vs. 154 ± 16; set 2: 160 ± 9 vs. 154 ± 11; set 3: 163 ± 10 vs. 158 ± 12; set 4: 162 ± 12 vs. 161 ± 9; set 5: 166 ± 9 vs. 163 ± 12); → Free –throw accuracy (%): (61.0 ± 17.0 vs. 55.0 ± 20.0).
Stojanović et al. (37)	10 F; 20 ± 4; Highly Trained; 9.4 + 3.2	< 100	Capsule; 3; 60	Placebo (dextrose)	SJ + LAD + CMJ + CMJAS + Sprint (0 −20 m) with and without dribbling test	↑10 m sprint (s): (2.0 ± 0.1 vs. 2.1 ± 0.2), 20 m sprint (s): (3.5 ± 0.2 vs. 3.6 ± 0.3); → 5 m sprint (s): (1.2 ± 0.1 vs. 1.2 ± 0.2), CMJ (cm): (29.2 ± 4.4 vs. 27.9 ± 4.2), CMJAS (cm): (35.1 ± 5.1 vs. 33.9 ± 3.9), SJ (cm): (27.2 ± 4.4 vs. 26.0 ± 3.2), LAD (s): (13.0 ± 0.9 vs. 13.2 ± 0.9), 5 m sprint with dribbling (s): (1.2 ± 0.1 vs. 1.2 ± 0.1), 10 m sprint with dribbling (s): (2.1 ± 0.1 vs. 2.1 ± 0.1), 20 m sprint with dribbling (s): (3.6 ± 0.3 vs. 3.7 ± 0.2).
Scanlan et al. (38)	11 M/10 F; 18 ± 3; Highly Trained; N.A.	< 100	Capsule; 3; 60	Placebo (dextrose)	Sprint (0 −20 m) with dribbling test	→ 5 m sprint with dribbling (s): (1.2 ± 0.1 vs.1.2 ± 0.1), 10 m sprint with dribbling (s): (2.0 ± 0.1 vs. 2.0 ± 0.1), 20 m sprint with dribbling (s): (3.5 ± 0.2 vs. 3.6 ± 0.2).
Puente et al. (39)	10 M/9 F; 28 ± 5; Elite; > 10	< 100	Capsule; 3; 60	Placebo (cellulose)	CODAT + AJ + SM test	↑Body impacts (number/min): (AA homozygotes: 401 ± 36 vs. 385 ± 48; C –allele carriers: 415 ± 35 vs. 401 ± 36), AJ (cm): (AA homozygotes: 40.7 ± 7.3 vs. 39.6 ± 7.2); → AJ (cm): (C –allele carriers: 37.2 ± 6.9 vs. 36.3 ± 5.9), Peak heart rate (bpm): (AA homozygotes: 188 ± 13 vs. 187 ± 12; C –allele carriers: 185 ± 6 vs. 182 ± 7), Mean heart rate (bpm): (AA homozygotes: 160 ± 10 vs. 158 ± 9; C –allele carriers: 163 ± 9 vs. 161 ± 13), CODAT with the ball (s): (AA homozygotes: 6.1 ± 0.2 vs. 6.2 ± 0.2; C –allele carriers: 6.1 ± 0.4 vs. 6.1 ± 0.4), CODAT without the ball (s): (AA homozygotes: 5.9 ± 0.3 vs. 5.9 ± 0.3; C –allele carriers: 6.0 ± 0.4 vs. 6.0 ± 0.3), Perceived muscle power: (AA homozygotes: 6.7 ± 1.3 vs. 5.3 ± 1.8; C –allele carriers: 6.2 ± 1.5 vs. 5.4 ± 0.9), Perceived endurance: (AA homozygotes: 6.8 ± 1.5 vs. 5.7 ± 1.6; C –allele carriers: 5.6 ± 1.7 vs. 5.6 ± 0.9).
Puente et al. (40)	10 M/10 F; 28 ± 5; Elite; > 10	< 100	Capsule; 3; 60	Placebo (cellulose)	AJ + CODAT + SM test	↑AJ (cm): (38.2 ± 7.4 vs. 37.3 ± 6.8), Body impacts: (410 ± 41 vs. 396 ± 43), Performance index: (11.6 ± 7.3 vs. 8.4 ± 8.3), Total rebounds: (3.7 ± 2.6 vs. 2.5 ± 2.0), Assists: (2.1 ± 1.6 vs. 1.1 ± 0.9); → CODAT without the ball (s): (6.0 ± 0.3 vs. 6.0 ± 0.3), CODAT with the ball (s): (6.1 ± 0.3 vs. 6.2 ± 0.3), Mean heart rate (bpm): (161 ± 10 vs. 157 ± 13), Peak heart rate (bpm): (188 ± 10 vs. 185 ± 12), Points: (8.8 ± 6.1 vs. 8.2 ± 6.9), Two –point shooting accuracy (%): (52.9 ± 37.2 vs. 54.7 ± 30.5), Three –point shooting accuracy (%): (23.7 ± 27.5 vs. 27.4 ± 31.5), Free –throw accuracy (%): (73.8 ± 20.7 vs. 71.4 ± 40.5), Turnovers: (1.7 ± 1.3 vs. 1.7 ± 1.5).
Cheng et al. (41)	15 M; 20 ± 2; Highly Trained; N.A.	50 −100	Capsule; 6; 60	Placebo (cellulose)	3 min Wingate test	↑Peak heart rate (bpm): (172 ± 7 vs. 165 ± 8).
Mahdavi et al. (42)	24 F; 24 ± 3; Highly Trained; N.A.	117 ± 27	Capsule; 5; 70	Placebo (dextrose)	30s Wingate test	→ Fatigue index (%): (45.2 ± 6.8 vs. 46.6 ± 6.8).
Abian –Vicen et al. (2)	16 F; 14 ± 0; Highly Trained; > 6	< 60	Energy drink; 3; 60	Placebo (decaffeinated drink)	CMJ + RJ −15 + Free –throw + Two –point shooting accuracy test +Yo–Yo IR −1 test	↑CMJ (cm): (38.3 ± 4.4 vs. 37.5 ± 4.4), RJ −15 (cm): (30.2 ± 3.6 vs. 28.8 ± 3.4) Perceived muscle endurance: (6.6 ± 1.4 vs. 5.1 ± 1.1), Perceived muscle power: (7.1 ± 1.1 vs. 5.2 ± 1.2); → Three –point shooting accuracy (%): (38.1 ± 12.8 vs. 39.9 ± 11.8), Free –throw accuracy (%): (70.3 ± 11.0 vs. 70.7 ± 11.8), Yo–Yo IR −1 test: (2000 ± 706 vs. 1925 ± 702).

→ , no change; ↑, increase; ↓, decrease.

AG, agility; AJ, abalakov jump; CMJ, countermovement jump; CMJAS, countermovement jump with arm swing; CODAT, change-of-direction and acceleration test; F, female; HDEC: high-dose espresso coffee; LDEC, low-dose espresso coffee; LAD, lane agility drill; M, male; N.A., not available; PIR, performance index rating.

Eleven studies reported basketball-specific skill outcomes (2, 18, 29, 31, 33, 34, 36–40). Thirteen studies assessed non-specific physical performance measures (2, 17–19, 29–31, 33–35, 37, 39, 40), while four studies evaluated performance during simulated basketball match play (19, 32, 39, 40). Additionally, ten studies reported physiological and perceptual responses measured during basketball-specific and non-specific tests, as well as during real or simulated competition (17, 30–33, 36, 37, 39, 40, 42).

### 3.2 Methodological quality

**Figure 2:**
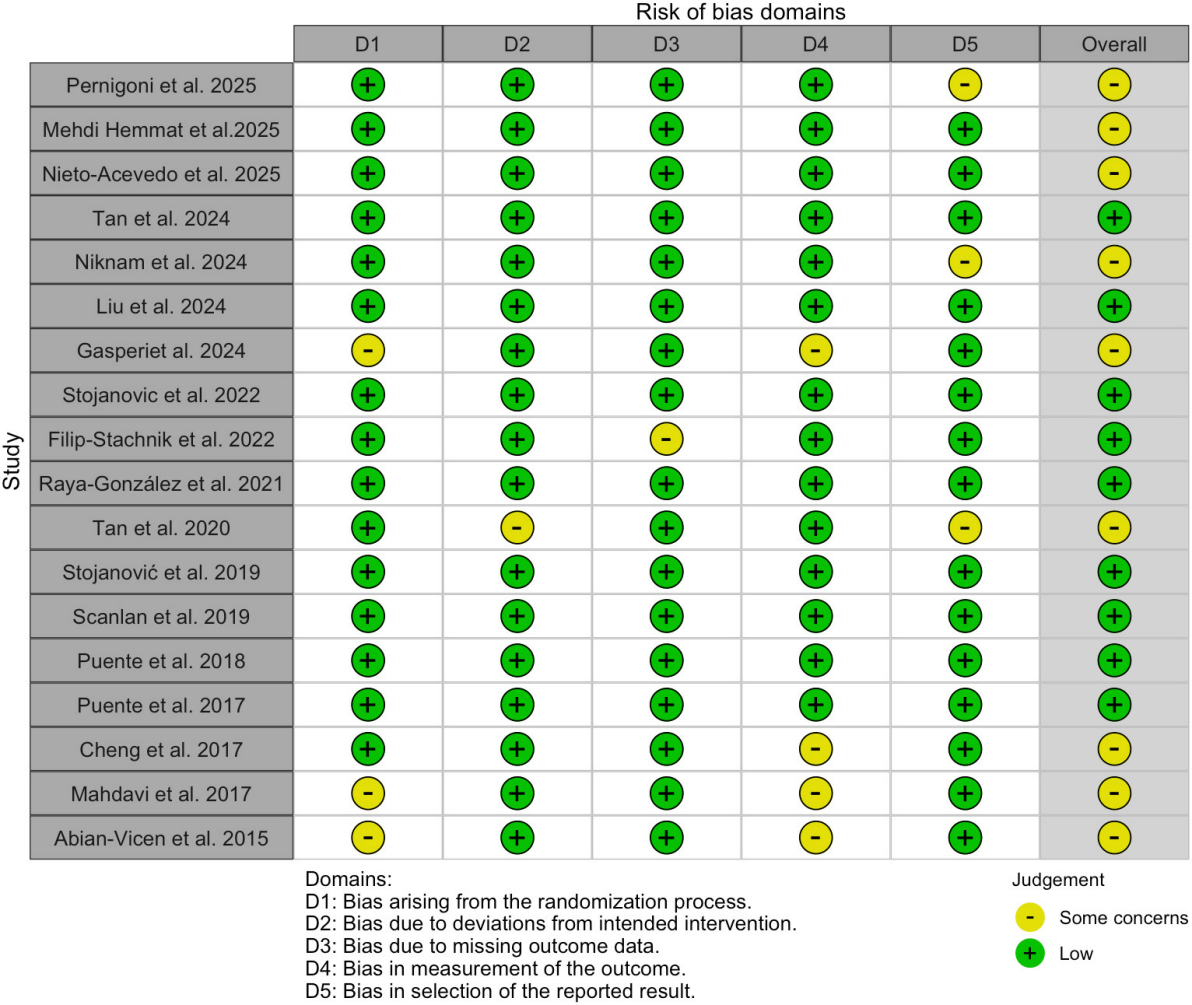
Risk-of-bias summary for the included studies.

Across the 18 included studies, nine were judged to have a *low risk* of bias, while 9 were rated as having *some concerns* (Figure 2). Funnel plot inspection revealed minor asymmetry, suggesting potential publication bias (Supplementary Figures S1–S8). Given the subjective nature of funnel plot interpretation, publication bias was further examined using Egger's tests, which showed no significant bias across the primary outcomes (all *p* > 0.05). In addition, meta-regression analysis showed no significant associations between effect sizes for all outcomes (e.g., specific and non-specific tests, game actions during real or simulated competition, and physiological and perceptual responses) and the independent variables caffeine doses and training years (all *p* > 0.05) (See Supplementary Table S1).

### 3.3 Meta-analysis results

#### 3.3.1 Basketball-specific outcomes

**Figure 3:**
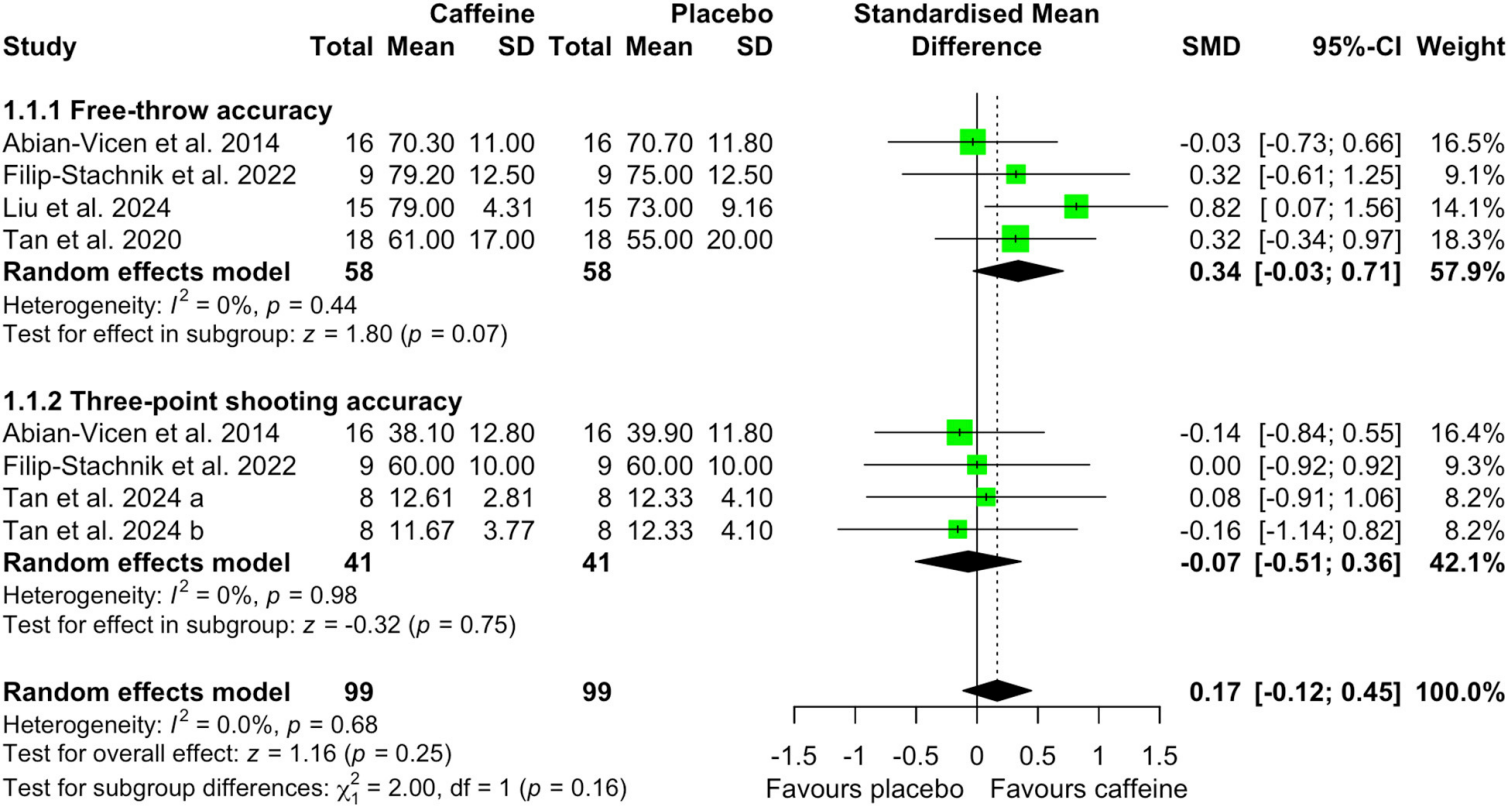
Effect of caffeine intake on free-throw and three-point shooting accuracy. Green squares represent the study-specific estimate, while diamonds indicate the pooled estimate from the random-effect model. CI: confidence interval; SD: standard deviation. “a”, “b”, represent the number of experiments in the same study, respectively.

**Figure 4:**
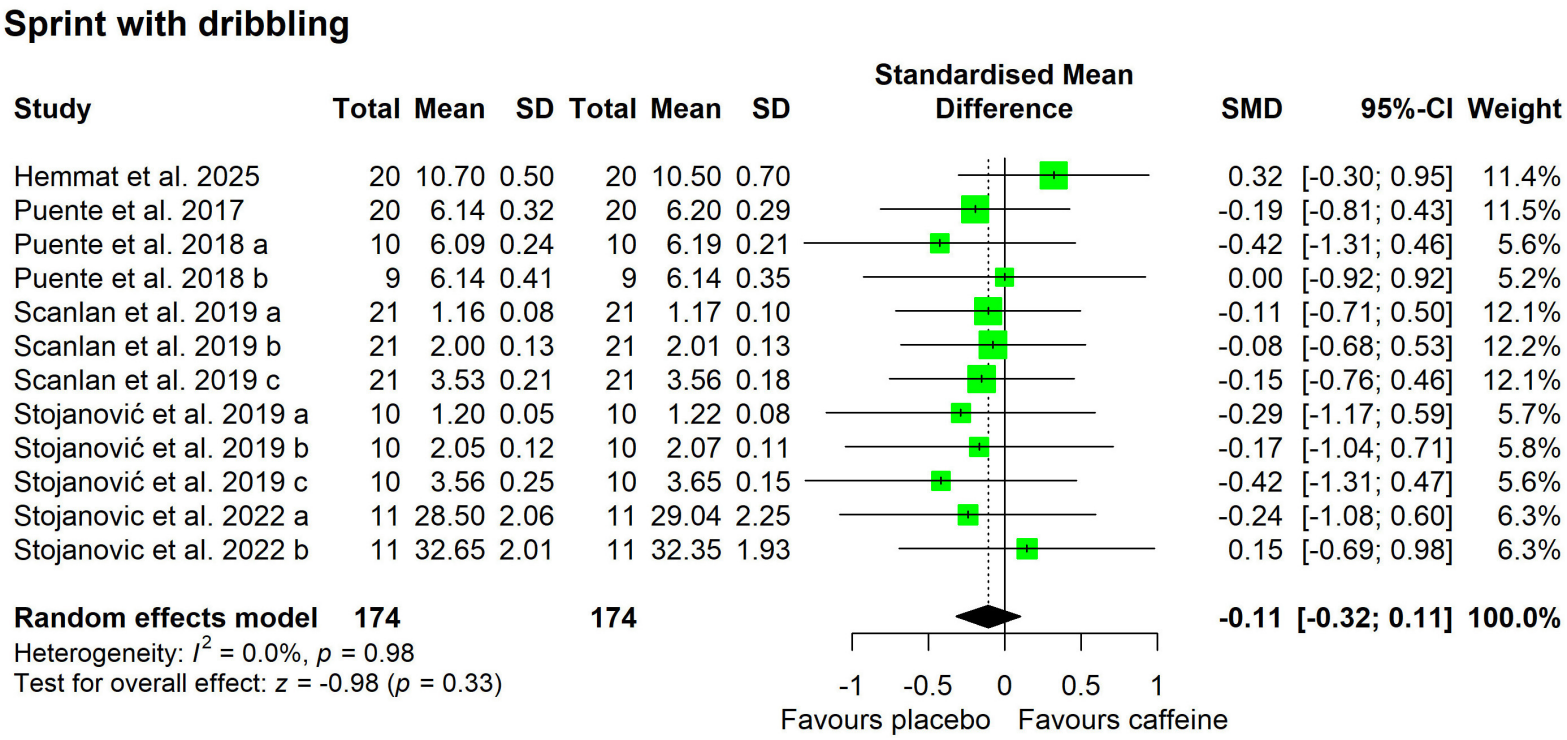
Effect of caffeine intake on sprint with dribbling. Green squares represent the study-specific estimate, while diamonds indicate the pooled estimate from the random-effect model. CI: confidence interval; SD: standard deviation. “a”, “b”, “c”, represent the number of experiments in the same study, respectively.

Compared with placebo, caffeine intake showed a tendency to improve free-throw accuracy (SMD = 0.34; 95% CI = −0.03 to 0.71; *p* = 0.07; *I*^2^ = 0%). Caffeine had no effect on three-point shooting accuracy (SMD = −0.07; 95% CI = −0.51 to 0.36; *p* = 0.75; *I*^2^ = 0%) or sprint performance with ball dribbling (SMD = −0.11; 95% CI = −0.32 to 0.11; *p* = 0.33; *I*^2^ = 0%) (Figures 3, 4).

#### 3.3.2 Non-specific outcomes

**Figure 5:**
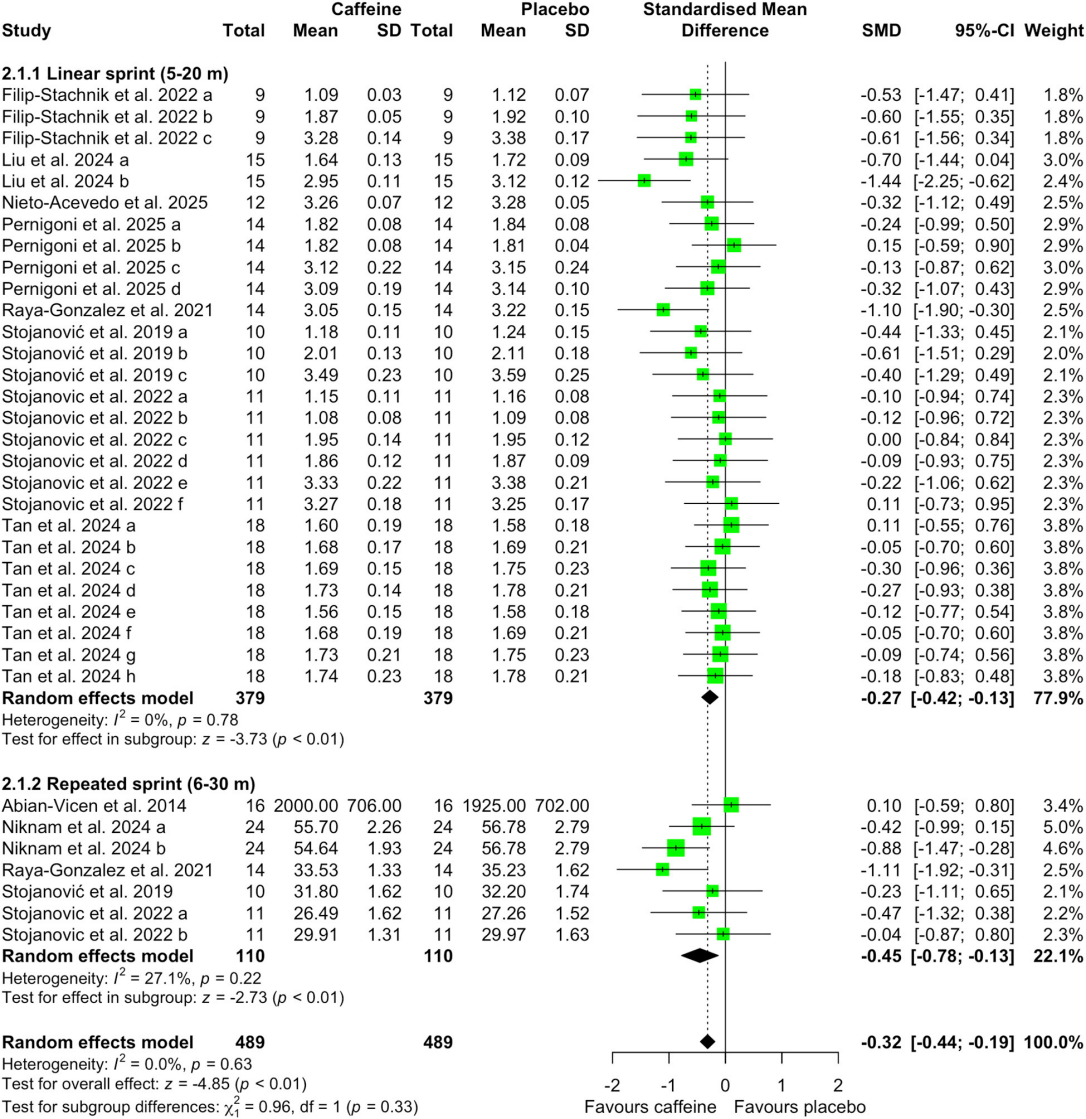
Effect of caffeine intake on sprint speed. Green squares represent the study-specific estimate, while diamonds indicate the pooled estimate from the random-effect model. CI: confidence interval; SD: standard deviation. “a”, “b”, “c”, “d”, “e”, “f”, “g”, “h”, represent the number of experiments in the same study, respectively.

**Figure 6:**
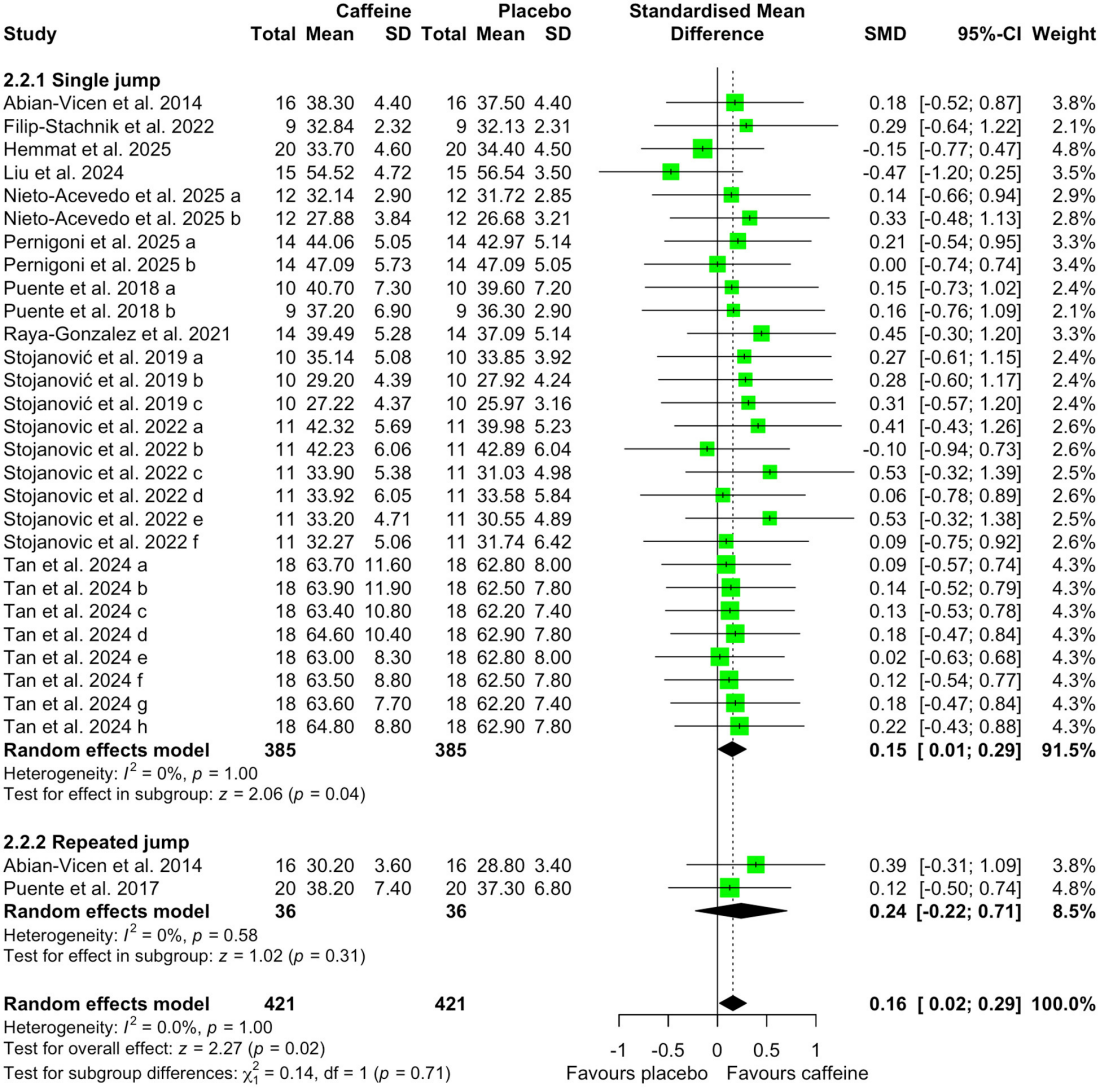
Effect of caffeine intake on single and repeated jump height. Green squares represent the study-specific estimate, while diamonds indicate the pooled estimate from the random-effect model. CI: confidence interval; SD: standard deviation. “a”, “b”, “c”, “d”, “e”, “f”, “g”, “h”, represent the number of experiments in the same study, respectively.

**Figure 7:**
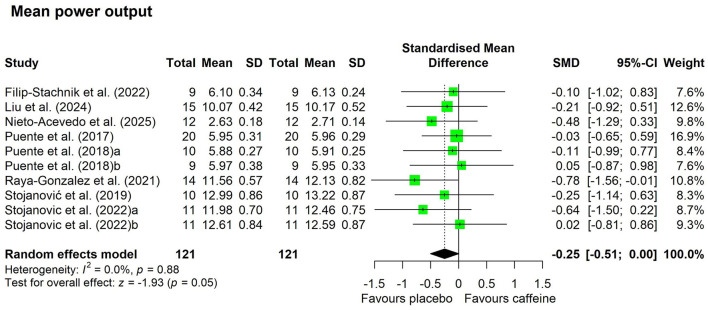
Effect of caffeine supplementation on mean power output. Effect of caffeine intake on agility. Green squares represent the study-specific estimate, while diamonds indicate the pooled estimate from the random-effect model. CI: confidence interval; SD: standard deviation. “a”, “b”, represent the number of experiments in the same study, respectively.

Figures 5–7 depict the effects of caffeine on non-basketball specific physical performance. Compared with placebo, caffeine intake reduced completion time in both linear sprint (5–20 m; SMD = −0.27; 95% CI = −0.42 to−0.13; *p* < 0.01; *I*^2^ = 0%) and repeated sprints (6–30 m; SMD = −0.45; 95% CI = −0.78 to−0.13; *p* < 0.01; *I*^2^ = 27%) (Figure 5). Furthermore, caffeine intake increased single jump height (SMD = 0.15; 95% CI = 0.01 to 0.29; *p* = 0.04; *I*^2^ = 0%) (Figure 6) and shortened agility test completion time (SMD = −0.25; 95% CI = −0.51 to 0.00; *p* = 0.05; *I*^2^ = 0%) (Figure 7), while showing no significant effect on repeated jump performance (SMD = 0.24; 95% CI = −0.22 to 0.71; *p* = 0.31; *I*^2^ = 0%) (Figure 6). In addition, caffeine intake showed no significant effect on sprint with dribbling performance (SMD = −0.11; 95% CI = −0.32 to 0.11; *p* = 0.33; *I*^2^ = 0%) and mean power output (SMD = −0.25; 95% CI = −0.51 to 0.00; *p* = 0.05; *I*^2^ = 0%) (Figure 7).

#### 3.3.3 Outcomes during simulated basketball competition

**Figure 8:**
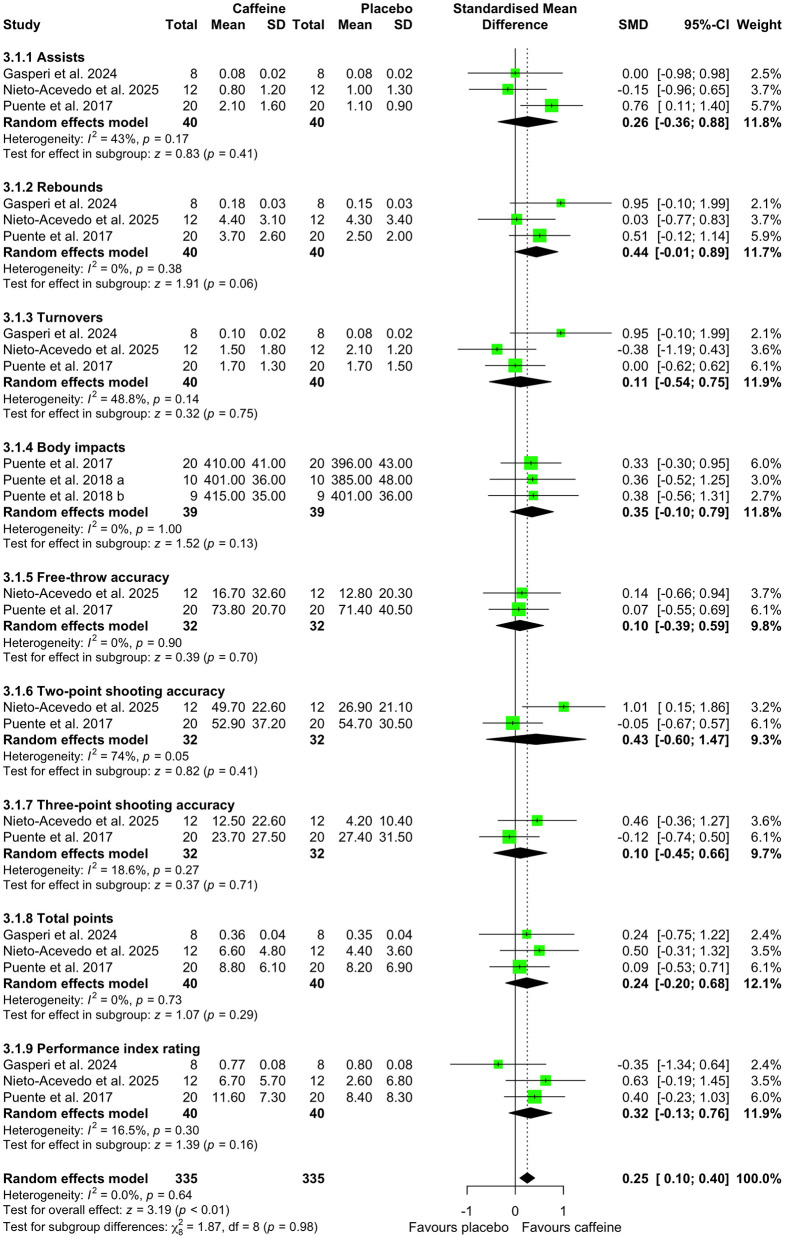
Effect of caffeine intake on overall basketball game performance. Green squares represent the study-specific estimate, while diamonds indicate the pooled estimate from the random-effect model. CI: confidence interval; SD: standard deviation. “a”, “b”, represent the number of experiments in the same study, respectively.

Meta-analysis of basketball-specific actions assessed during real or simulated competition indicated that caffeine intake increased overall basketball game performance compared with placebo (SMD = 0.25; 95% CI = 0.10 to 0.40; *p* < 0.01; *I*^2^ = 0%). Subgroup analyses revealed no significant effects of caffeine on individual game actions, including assists (SMD = 0.26; 95% CI = −0.36 to 0.88; *p* = 0.41; *I*^2^ = 43%), rebounds (SMD = 0.44; 95% CI = −0.01 to 0.89; *p* = 0.06; *I*^2^ = 0%), turnovers (SMD = 0.11; 95% CI = −0.54 to 0.75; *p* = 0.75; *I*^2^ = 49%), body impacts (SMD = 0.35; 95% CI = −0.10 to 0.79; *p* = 0.13; *I*^2^ = 0%), free-throw accuracy (SMD = 0.10; 95% CI = −0.39 to 0.59; *p* = 0.70; *I*^2^ = 0%), two-point shooting accuracy (SMD = 0.43; 95% CI = −0.60 to 1.47; *p* = 0.41; *I*^2^ = 74%), three-point shooting accuracy (SMD = 0.10; 95% CI = −0.45 to 0.66; *p* = 0.71; *I*^2^ = 19%), and total points (SMD = 0.24; 95% CI = −0.20 to 0.68; *p* = 0.29; *I*^2^ = 0%), and performance index rating (SMD = 0.32; 95% CI = −0.13 to 0.76; *p* = 0.30; *I*^2^ = 17%) (Figure 8).

#### 3.3.4 Physiological and perceptual responses

**Figure 9:**
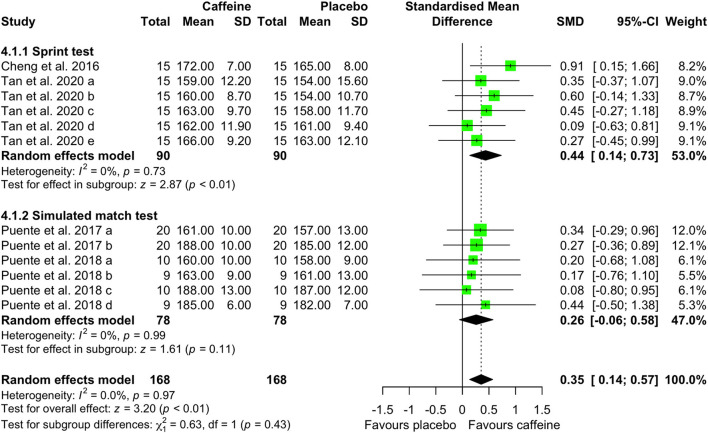
Effect of caffeine intake on heart rate. Green squares represent the study-specific estimate, while diamonds indicate the pooled estimate from the random-effect model. CI: confidence interval; SD: standard deviation. “a”, “b”, “c”, “d”, “e”, represent the number of experiments in the same study, respectively.

**Figure 10:**
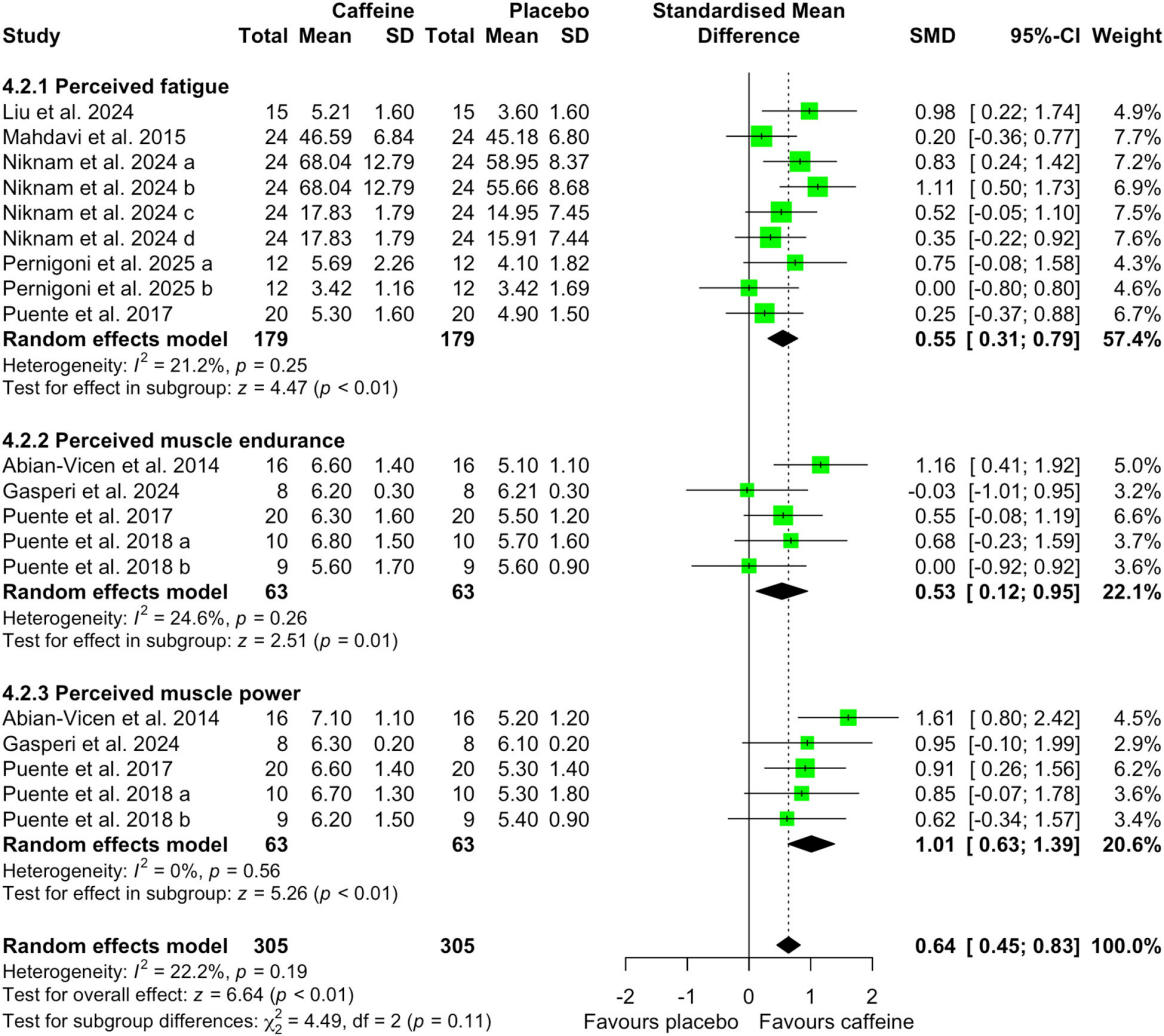
Effect of caffeine intake on perceived fatigue, muscle endurance, and power. Green squares represent the study-specific estimate, while diamonds indicate the pooled estimate from the random-effect model. CI: confidence interval; SD: standard deviation. “a”, “b”, “c”, represent the number of experiments in the same study, respectively.

Compared with placebo, caffeine intake significantly increased heart rate (SMD = 0.35; 95% CI = 0.14 to 0.57; *p* < 0.01; *I*^2^ = 0%) during sprint-based testing, whereas no significant effect was observed during simulated match play (SMD = 0.26; 95% CI = −0.06 to 0.58; *p* = 0.11; *I*^2^ = 0%) (Figure 9). Caffeine also had significant effects on perceptual responses. Specifically, perceived power (SMD = 1.01; 95% CI = 0.63 to 1.39; *p* < 0.01; *I*^2^ = 0%) and perceived endurance (SMD = 0.53; 95% CI = 0.12 to 0.95; *p* = 0.01; *I*^2^ = 25%) were increased following caffeine intake, with a decrease in perceived fatigue (SMD = 0.55; 95% CI = 0.31 to 0.79; *p* < 0.01; *I*^2^ = 21%) (Figure 10).

## 4 Discussion

### 4.1 Summary of main findings

This meta-analysis examined how the well-documented ergogenic effects of caffeine on physical performance extend to basketball-specific actions assessed during isolated skill tests and competitive game scenarios. The main findings indicate that pre-exercise low-to-moderate doses of caffeine enhanced general physical capacities relevant to basketball (i.e., sprint speed, jump height, and agility). In contrast, low caffeine doses did not meaningfully improve isolated skills (i.e., free-throw and three-point shooting accuracy, dribbling speed). However, low doses of caffeine were associated with superior overall performance during real or simulated competitions, alongside heightened perceptions of muscle endurance and power. Collectively, these findings indicate that low caffeine doses primarily enhance basketball performance by supporting the physical and perceptual demands of match play rather than by directly enhancing discrete technical skills.

### 4.2 Effects of caffeine intake on non-basketball-specific performance

Basketball performance relies on the repeated execution of high-intensity actions, including speed, agility, anaerobic capacity, and vertical jump ability, all of which place substantial demands on anaerobic power and neuromuscular function (1, 45). Match play analyses indicate that players perform more than 50 jumps per game (46), complete a sprint approximately every 39s [48], and that ~52% of sprinting actions involve curved running paths and frequent changes of direction (47). Our results demonstrated that caffeine intake significantly enhanced linear (5–20 m) and repeated (6–30 m) sprint speed (Figure 5), increased single-jump height (Figure 6), and improved agility outcomes (Figure 7). Comparable ergogenic effects have been reported in other team sports, including volleyball, with documented improvements in jump ability and agility following caffeine intake (14). These physical performance benefits are likely mediated by well-established neuromuscular mechanisms, including increased neurotransmitter release (e.g., β-endorphins and catecholamines) (48), intracellular calcium availability (10), and antagonism of adenosine receptors within the central nervous system (11, 49). Collectively, these mechanisms may enhance muscular power output. This interpretation is supported by the observed elevations in heart rate during sprint-based tasks following caffeine intake (Figure 9), suggesting heightened physiological activation during high-intensity efforts.

### 4.3 Effects of caffeine intake on basketball-specific and game performance

A key contribution of this meta-analysis was the examination of the effects of caffeine intake on basketball-specific skills, such as shooting accuracy and dribbling speed. Overall, caffeine intake did not meaningfully improve free-throw or three-point shooting accuracy, nor dribbling speed (Figures 3–4). These observations suggest that the ergogenic benefits of caffeine observed in basketball are unlikely to directly translate to isolated technical skill execution. Several factors may explain these null effects. Most notably, the caffeine doses administered across studies were relatively low, particularly in relation to participants' habitual caffeine consumption. Among the five studies assessing shooting accuracy, four employed low caffeine doses (2.3–3 mg/kg), while participants were generally classified as mild habitual caffeine consumers (74–200 mg/day, 0.96–2.71 mg/kg/day) (2, 29, 31, 34, 36). This dosing strategy may have limited the potential for an acute ergogenic response. Supporting this, a previous meta-analysis in volleyball players demonstrated that moderate caffeine doses (3–6 mg/kg) significantly improved attacking and serving accuracy (14). Moreover, habitual caffeine consumption may blunt the ergogenic effects of acute caffeine intake (50, 51). As suggested by Pickering and Kiely (52), this attenuation may be mitigated when acute caffeine doses exceed an individual's habitual daily intake. Collectively, these findings suggest that caffeine doses greater than 3 mg/kg may be required to elicit measurable improvements in basketball shooting performance, particularly in players who regularly consume caffeine.

Several contextual and methodological factors may help explain the mixed effects of caffeine on basketball-specific performance outcomes. First, participants' training status may have influenced the observed outcomes. Athletes included in the reviewed studies were classified as highly trained to elite, and the ergogenic effects of caffeine are known to vary with training status (38, 40). Highly trained individuals may have limited scope for further performance enhancement following caffeine ingestion, as they already operate close to their maximal performance potential (53, 54). Second, variability in shooting assessment protocols may contributed to the inconsistent findings. In three studies reporting no improvements in free-throw accuracy following caffeine intake (2, 34, 36), the number of attempts was relatively low (10–12 attempts), compared with the study demonstrating a positive effect (31), which employed 30 attempts. A limited number of trials may reduce statistical sensitivity and the ability to detect meaningful differences. Together, these factors highlight the importance of considering both athlete characteristics and test design when interpreting the effects of caffeine on basketball-specific skill performance.

Another key contribution of our analysis was the evaluation of the effects of caffeine intake on performance during real or simulated basketball competition. Although caffeine intake did not significantly influence individual technical indicators during match play, it was associated with a small but meaningful improvement in overall game performance (Figure 8). This dissociation suggests that caffeine's ergogenic effects in basketball may manifest at the level of global performance rather than through isolated technical actions. Potential mechanisms underlying these effects include central and peripheral pathways. First, caffeine acts on adenosine receptors in the brain, thereby enhancing attentional, alertness, and cognitive function (55), which may support decision-making and sustained engagement during gameplay (56). Second, caffeine-induced increases in endorphin concentration may attenuate pain perception (57), enabling a higher rate of work at a given level of perceived discomfort and thereby reducing fatigue over the course of the game (56). Third, caffeine may increase motor unit recruitment (58) and muscle activation (59), thereby enhancing the ability to generate greater strength and power. These postulated mechanisms are consistent with our findings of decreased perceived fatigue alongside increased perceived endurance and power (Figures 9, 10). It should be noted, however, that most included studies employed a relatively low caffeine dose (3 mg/kg). Consequently, future research should further examine the feasibility, safety, and potential performance benefits of higher caffeine dose (>3 mg/kg) during real or simulated basketball competition to better delineate dose-response relationships in ecologically valid settings.

### 4.4 Limitations and future considerations

This study has several limitations. First, the findings should be interpreted with caution because most participants were male, with female athletes representing only 37% of the total sample. Previous meta-analyses report inconsistent findings in female athletes. For instance, Gomez-Burton et al. (60) observed improvements in countermovement jump and handgrip strength but not in repeated sprint ability, while Wang et al. (61) reported enhanced repeated sprint ability in both sexes without significant sex differences. These inconsistencies may partly reflect female-specific factors, such as menstrual cycle–related hormonal fluctuations and oral contraceptive use. Hormonal variations across the menstrual cycle may influence neuromuscular function and substrate utilization, potentially attenuating caffeine's ergogenic effects (62, 63), while oral contraceptives may slow caffeine metabolism through CYP1A_2_ inhibition (64). Therefore, future studies should report menstrual cycle phase and oral contraceptive use and, where possible, conduct placebo and caffeine trials within the same menstrual cycle phase to minimize hormonal influences. In addition, examining potential sex differences in the ergogenic effects of caffeine in basketball players is warranted.

Second, only three included studies examined caffeine intake during real or simulated basketball match play (19, 32, 40). In one study, substitution frequency was restricted to equalize playing time during simulated games (40), potentially increasing physiological demands compared with official competition. Additionally, differences in simulated match protocols may have contributed to the substantial heterogeneity observed in two-point shooting accuracy outcomes (Figure 8). Consequently, further research is required to evaluate the effects of caffeine under ecologically valid, real-world competitive conditions. Third, although placebo and caffeine conditions were matched for all ingredients except caffeine, the potential additive or synergistic effects of caffeine when co-ingested with other ergogenic substances (e.g., taurine) cannot be fully excluded. Additionally, dietary intake and the use of other ergogenic aids were not consistently reported or controlled across the included studies, which may also have influenced the observed performance outcomes. Future studies should aim to better control these effects to isolate the independent effects of caffeine supplementation.

## 5 Conclusion

In basketball, low-dose caffeine intake appears to primarily enhance the physical and perceptual demands underpinning match play, with consistent improvements observed in non-specific performance measures and perceptual responses. These effects translate into improved overall game performance in real or simulated contexts, despite limited effects on isolated technical skills. Taken together, higher caffeine dosage (>3 mg/kg) may be required to elicit broader performance benefits, likely by further supporting physical capacity and perceptual regulation during competition. However, these findings should be interpreted with caution, as most participants were male, limiting the generalizability to female athletes. Moreover, practical application should consider moderating factors such as training status, individual habitual caffeine intake, and caffeine form. These variables may influence both performance and perceptual responses, and should be accounted for to better define caffeine's true effects on basketball performance and strengthen evidence-based recommendations.

## Data Availability

The original contributions presented in the study are included in the article/supplementary material, further inquiries can be directed to the corresponding authors.
