# Supplementary material for: Effects of caffeine intake on exercise performance in basketball players: a systematic review and meta-analysis
Source: Front Nutr. 2026 May 7;13:1837912. doi: 10.3389/fnut.2026.1837912 (PMC13191373; doi:10.3389/fnut.2026.1837912)
Supplement: Supplementary file 1 [file Supplementary_file_1.docx]

Title: Effects of Caffeine Intake on Exercise Performance in Basketball Players: A Systematic Review and Meta-Analysis

Journal: Frontiers in Nutrition

Authors: Jingxin Liu^1,†^, Ruiguo Xue^2,†^, Xuanjia Zhang^1,†^, Jin Huang^2^, Bin Chen^3^, Li Guo^4^, Yinhang Cao^2,*^, Olivier Girard^5^

1 School of Physical Education, Shanghai University of Sport, Shanghai, China

2 School of Athletic Performance, Shanghai University of Sport, Shanghai, China

3 Department of Public Physical Education, Fujian Agriculture and Forestry University, Fuzhou, China

4 School of Exercise and Health, Shanghai University of Sport, Shanghai, China

5 School of Human Sciences (Exercise and Sport Science), The University of Western Australia, Perth, Australia

† These authors contributed equally to this work.

*Correspondence:
Yinhang Cao

School of Athletic Performance

Shanghai University of Sport

Shanghai City 200438, China

Tel & Fax: +86-21-65507125

E-mail: [caoyinhang@sus.edu.cn](mailto:caoyinhang@sus.edu.cn)

**
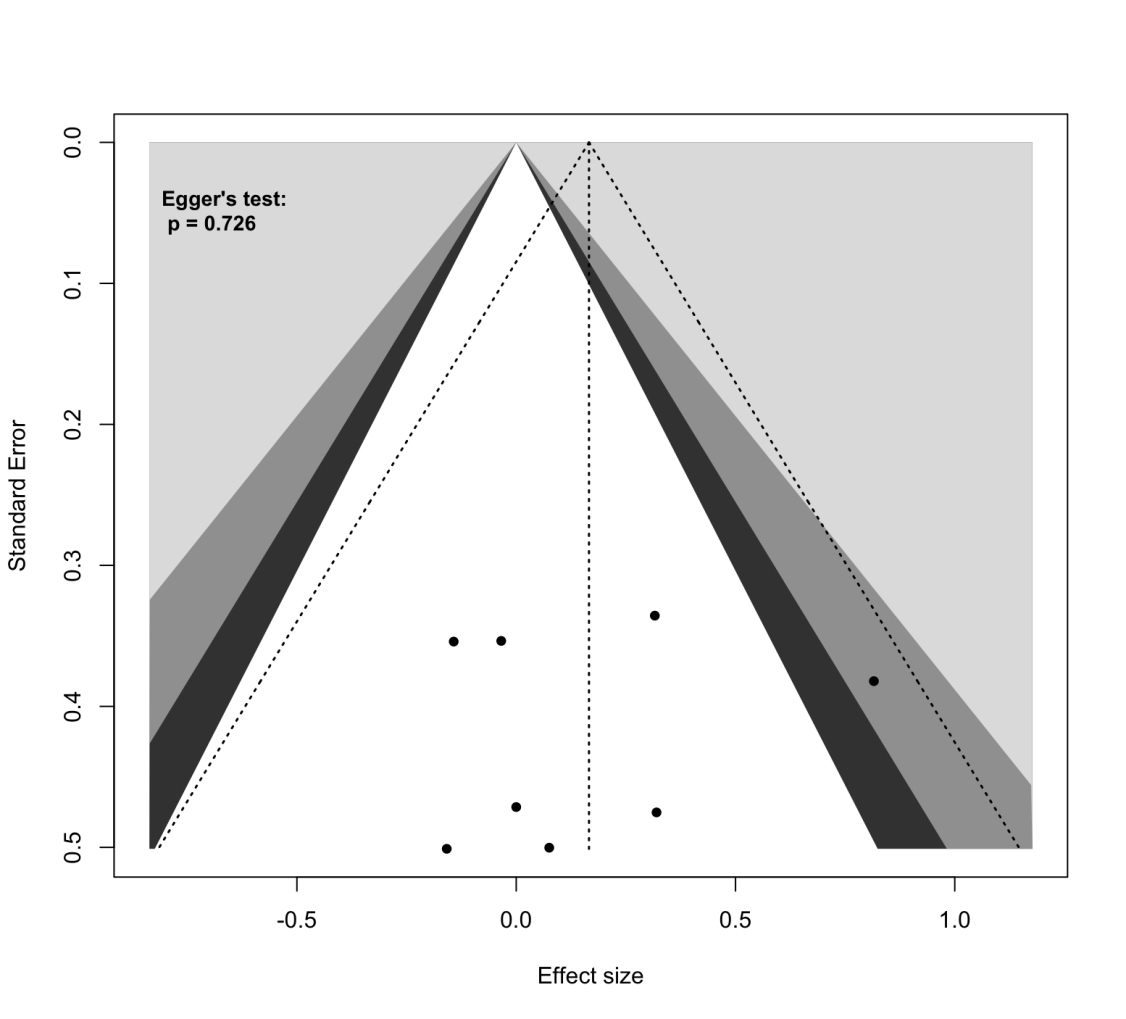
**

**Fig. S1** Funnel plot and the Egger's test results on free-throw and three-point shooting accuracy.


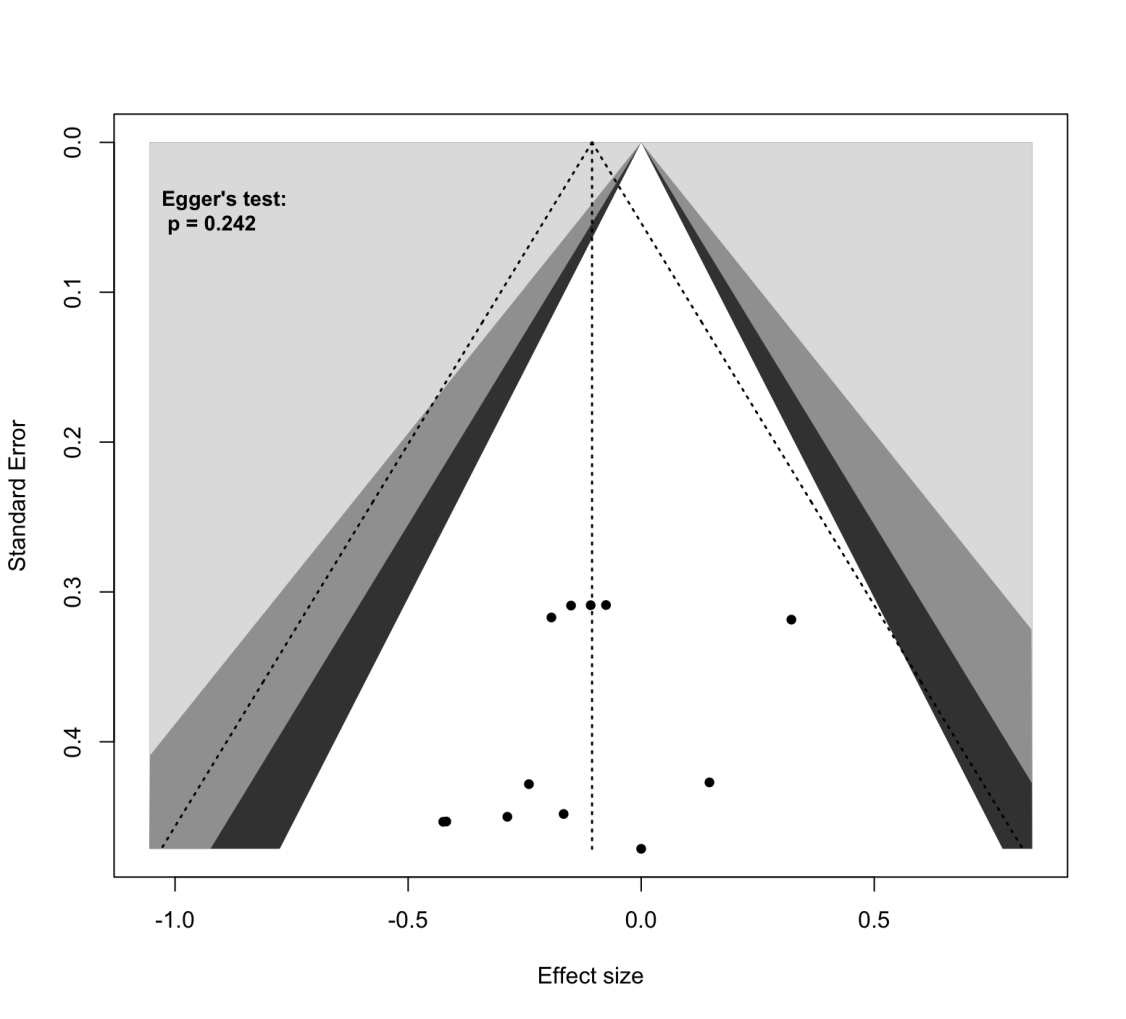


**Fig. S2** Funnel plot and the Egger's test results on sprint with dribbling.


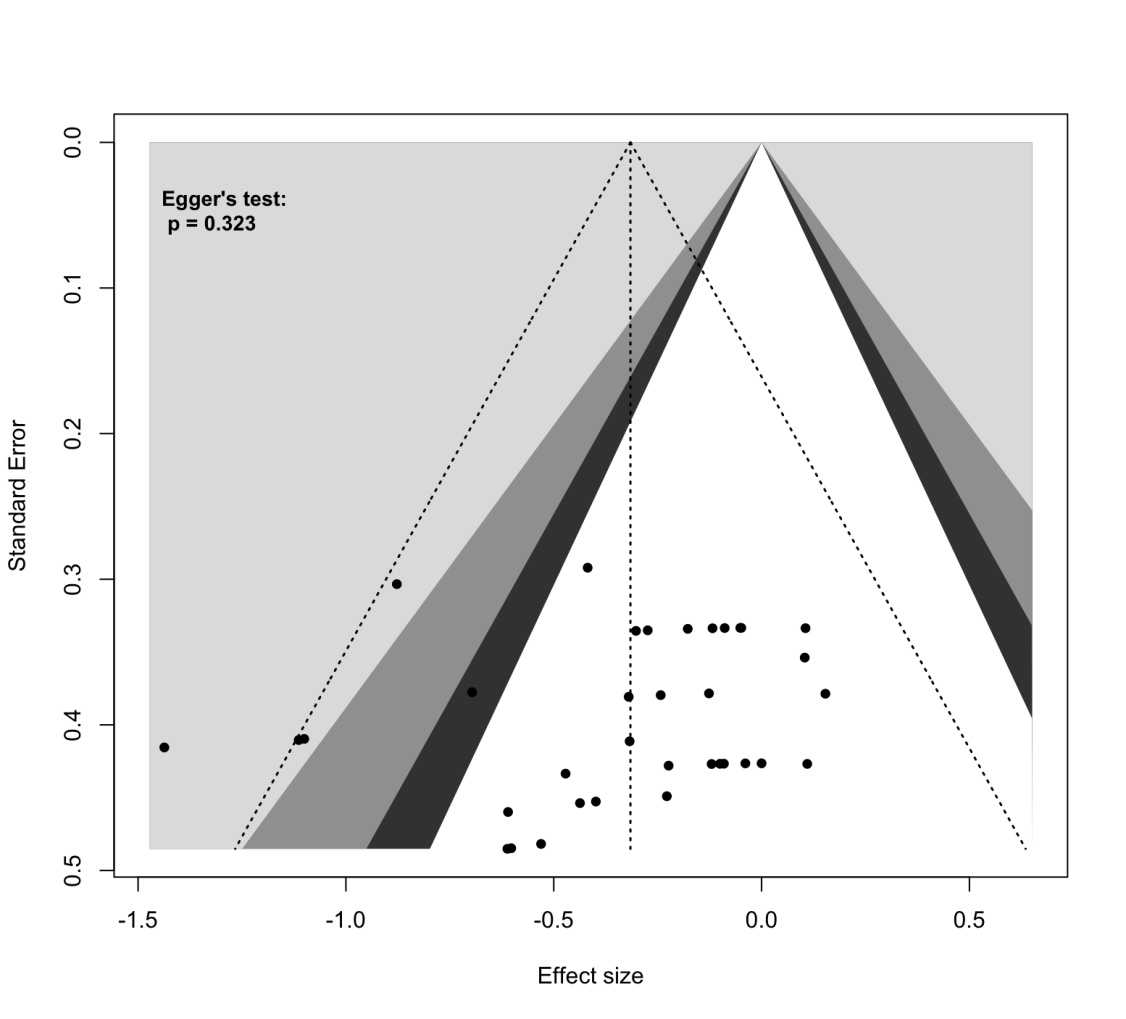


**Fig. S3** Funnel plot and the Egger's test results on linear and repeated sprint.


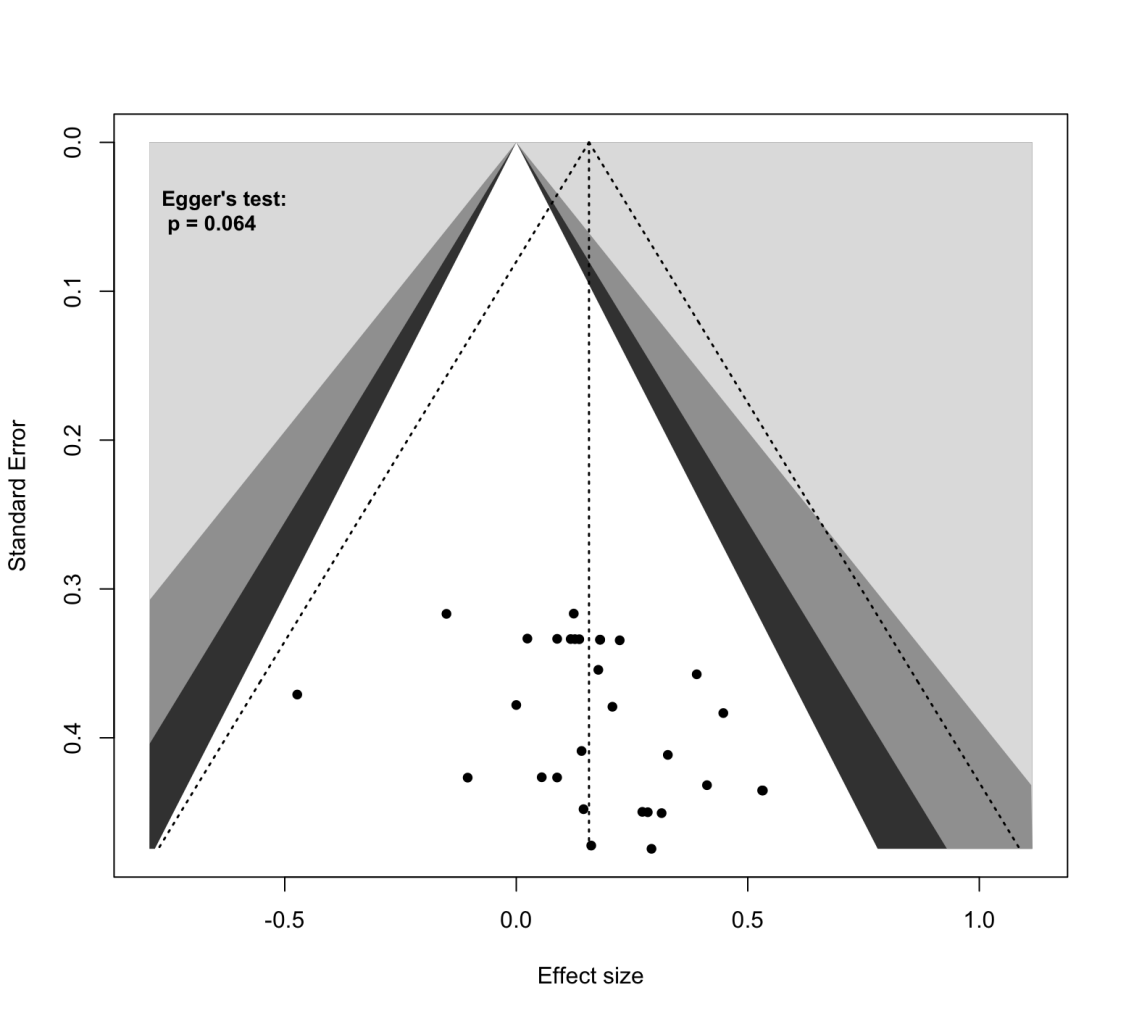


**Fig. S4** Funnel plot and the Egger's test results on single and repeated jump.


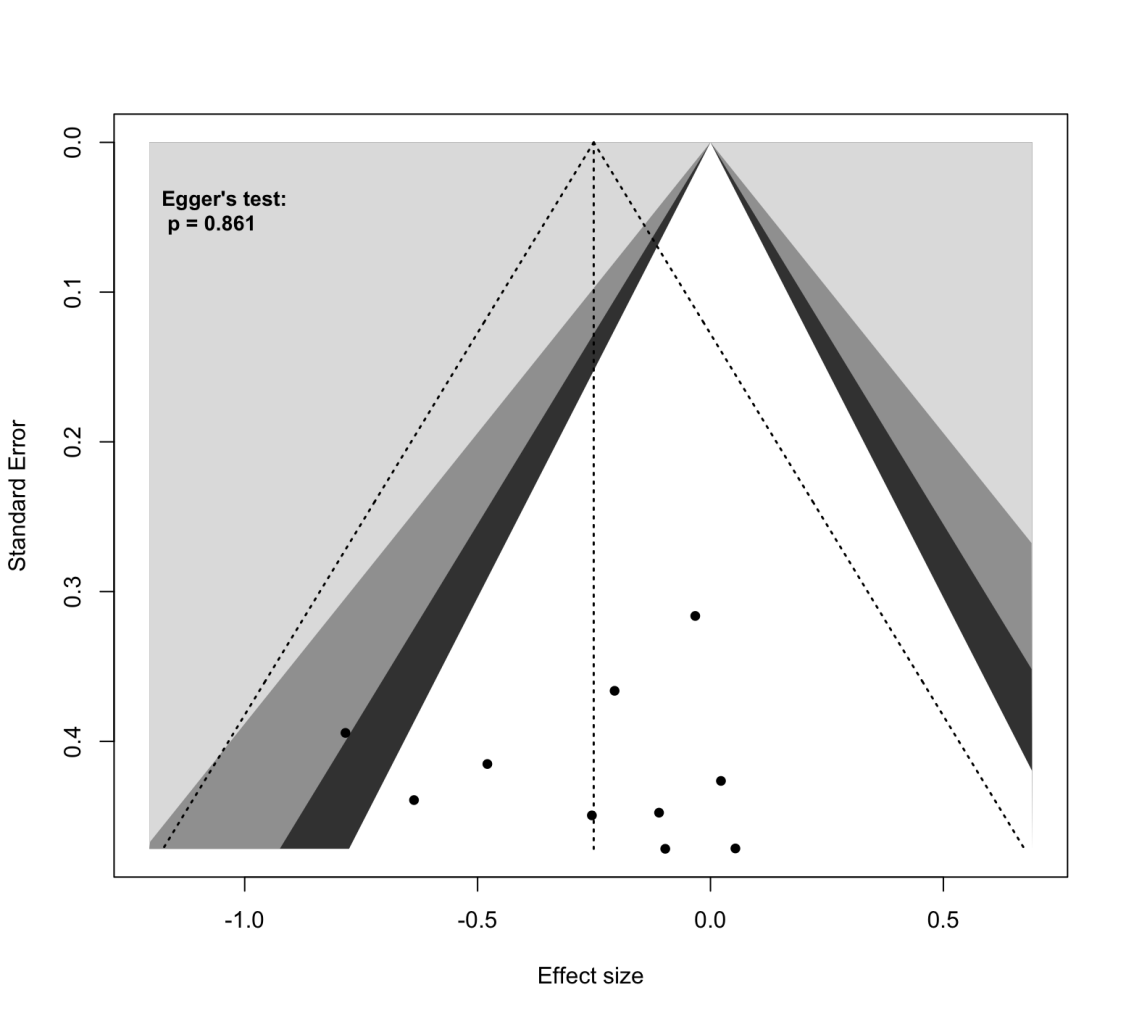


Fig. S5 Funnel plot and the Egger's test results on agility.


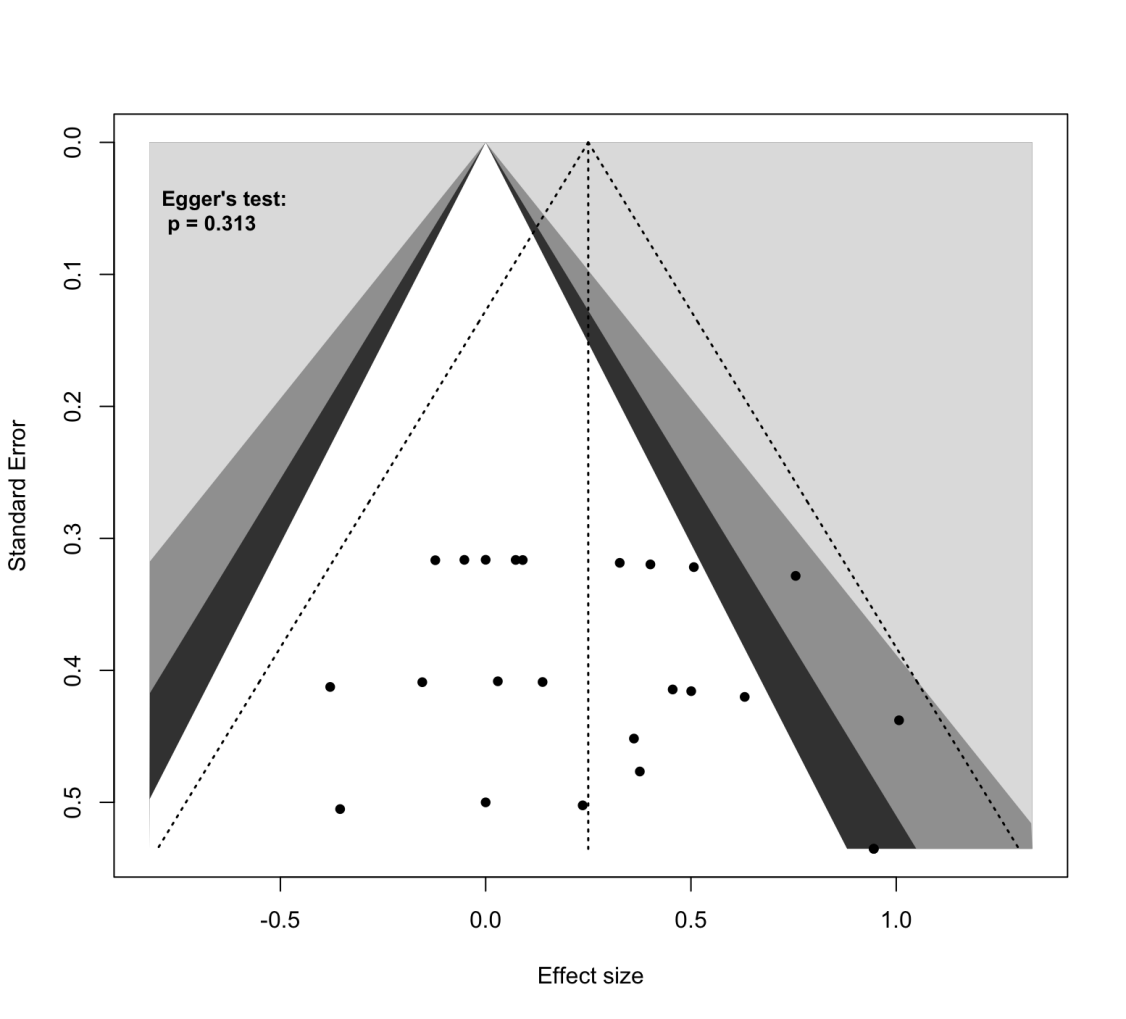


Fig. S6 Funnel plot and the Egger's test results on game actions during real or simulated competition.


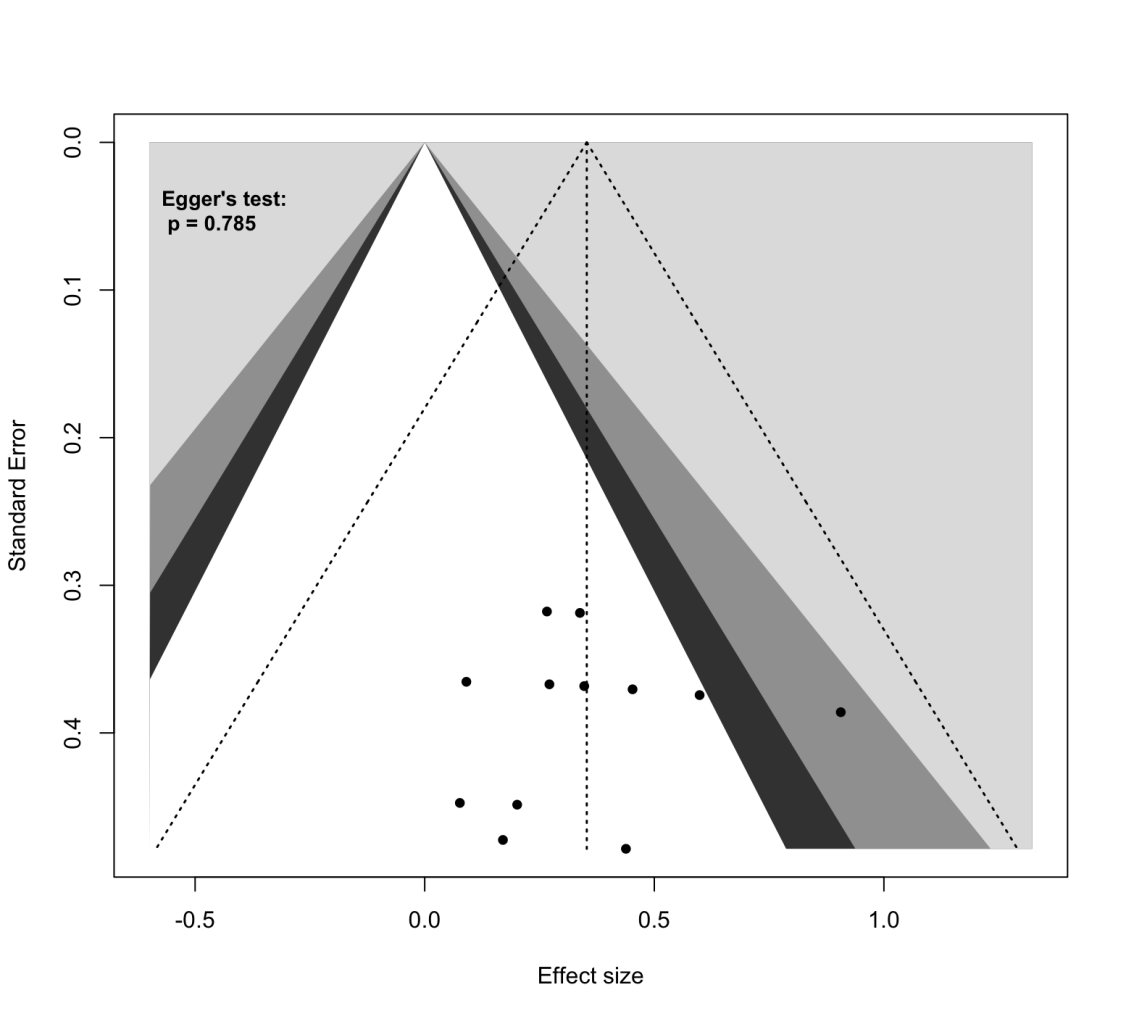


**Fig. S7** Funnel plot and the Egger's test results on heart rate.


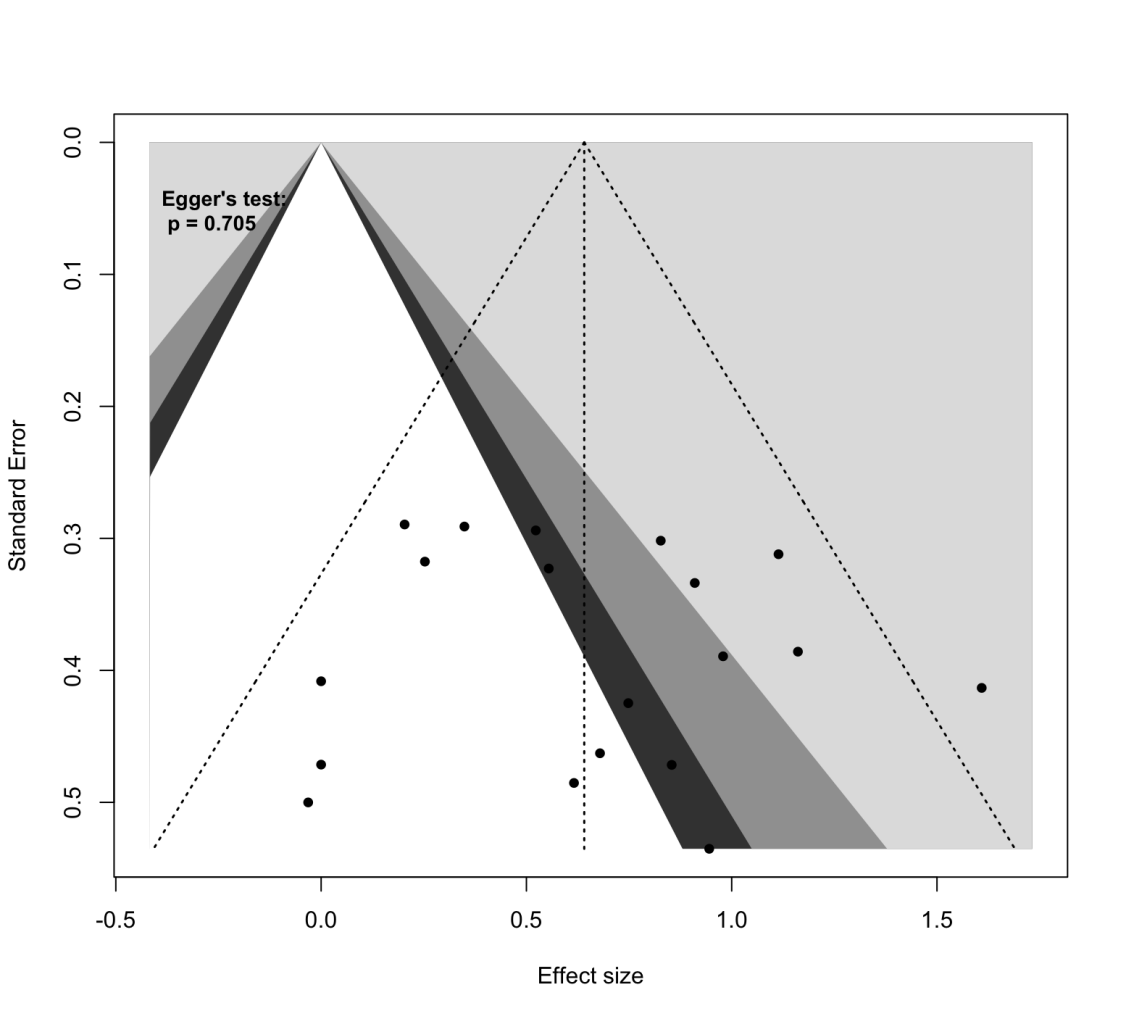


**Fig. S8** Funnel plot and the Egger's test results on perceived fatigue, endurance, and power.

Table S1 Meta-regression analysis

| Variables | Outcomes indicators | | Estimate | P | 95%CI | |
| --- | --- | --- | --- | --- | --- | --- |
|  |  |  |  |  | Lower Limit | Upper Limit |
| Dose | Basketball-specific outcomes | Free-throw and three-point shooting accuracy | 0.0534 | 0.6422 | -0.1719 | 0.2786 |
|  |  | Sprint with dribbling | - | - | - | - |
|  | Non-specific outcomes | Sprint speed | -0.1150 | 0.1428 | -0.2687 | 0.0388 |
|  |  | Single and repeated jump height | 0.0888 | 0.4847 | -0.1602 | 0.3377 |
|  |  | Agility | -0.1933 | 0.1491 | -0.4560 | 0.0693 |
|  | Outcomes during simulated basketball competition | Overall basketball game performance | - | - | - | - |
|  | Physiological and perceptual responses | Heart rate | 0.0585 | 0.4277 | -0.0861 | 0.2031 |
|  |  | Perceived fatigue, muscle endurance, and power. | -0.0965 | 0.3526 | -0.2999 | 0.1069 |
| Training years | Basketball-specific outcomes | Free-throw and three-point shooting accuracy | 0.0257 | 0.6049 | -0.0716 | 0.1229 |
|  |  | Sprint with dribbling | -0.0714 | 0.1400 | -0.1661 | 0.0234 |
|  | Non-specific outcomes | Sprint speed | -0.0133 | 0.4706 | -0.0493 | 0.0228 |
|  |  | Single and repeated jump height | 0.0092 | 0.6657 | -0.0326 | 0.0510 |
|  |  | Agility | 0.0484 | 0.4334 | -0.0727 | 0.1695 |

Table S1. (Continued).

|  | Outcomes during simulated basketball competition | Overall basketball game performance | 0.0245 | 0.7257 | -0.1126 | 0.1616 |
| --- | --- | --- | --- | --- | --- | --- |
|  | Physiological and perceptual responses | Heart rate | 0.0007 | 0.9625 | -0.0284 | 0.0298 |
|  |  | Perceived fatigue, muscle endurance, and power. | -0.0335 | 0.1837 | -0.0830 | 0.0159 |
